# Imaging Sub-Cellular Methionine and Insulin Interplay in Triple Negative Breast Cancer Lipid Droplet Metabolism

**DOI:** 10.3389/fonc.2022.858017

**Published:** 2022-03-10

**Authors:** Anthony A. Fung, Khang Hoang, Honghao Zha, Derek Chen, Wenxu Zhang, Lingyan Shi

**Affiliations:** Department of Bioengineering, University of California San Diego, La Jolla, CA, United States

**Keywords:** stimulated Raman scattering, heavy water, TPF, lipid metabolism, methionine, insulin, breast cancer, DO-SRS

## Abstract

Triple negative breast cancer (TNBC) is a particularly aggressive cancer subtype that is difficult to diagnose due to its discriminating epidemiology and obscure metabolome. For the first time, 3D spatial and chemometric analyses uncover the unique lipid metabolome of TNBC under the tandem modulation of two key metabolites – insulin and methionine - using non-invasive optical techniques. By conjugating heavy water (D_2_O) probed Raman scattering with label-free two-photon fluorescence (TPF) microscopy, we observed altered *de novo* lipogenesis, 3D lipid droplet morphology, and lipid peroxidation under various methionine and insulin concentrations. Quantitative interrogation of both spatial and chemometric lipid metabolism under tandem metabolite modulation confirms significant interaction of insulin and methionine, which may prove to be critical therapeutic targets, and proposes a powerful optical imaging platform with subcellular resolution for metabolic and cancer research.

## Introduction

Breast cancer is the most reported form of cancer in biological women, but the pathophysiology is rife with subtypes that have material consequences on patient outcomes. Triple negative breast cancer (TNBC) is a particularly aggressive cancer subtype that accounts for approximately 15% of all breast cancer cases and its epidemiology reveals a discriminating predilection for non-Hispanic African women (1, 2) (**Figure S1**). Although the genomes and proteomes of these breast cancer subtypes are distinguishable, little is known about their metabolic phenotypes and the consequential prognoses they manifest.

Recently, lipid metabolism has emerged as a major indicator of cellular stress, phenotypic state, and disease status in biological research and medicine. Dysregulation of lipid metabolism and heightened lipid synthesis are hallmarks of cancer, as varying demands of lipids for energy maintenance, metastasis, and angiogenesis warrant transcriptional changes that contribute to the metabolic phenotype (3–5). The quantity and diversity of lipids and their functions have been instrumental in profiling cancers as well. For example, membrane lipid compositions of cholesterol, phosphatidylcholine (PC), and phosphatidylethanolamine (PE) are essential to cell membrane fluidity, which has become a target for cancer treatments (6–9). Additionally, the degree of saturation of lipid content in a cell may provide further insight into its state of stress, as breast cancer cells may produce more saturated and monounsaturated membrane lipids to guard against oxidative stress (10–12). To interrogate lipid metabolism, lipid droplets (LD) were the primary organelle of interest since their ubiquitous structures not only serve as energy stores, but are also involved in protein folding and trafficking, signaling pathways, and have diverse spatial and chemical information that may reflect oxidative stress, metabolic flux, and disease status (10–18). However, direct visualization of LD metabolism manipulated by tandem nutritional interventions at a subcellular level has not yet been reported in TNBC cells, which is partially due to a lack of spatial resolution in conventional lipidomic modalities. Optical techniques such as spontaneous Raman spectroscopy and SRS imaging microscopy are well suited to both the chemometric and spatial dimensions for imaging LD metabolism; they can analyze not only the size, number, and distribution of LDs, but also their protein and lipid diversity and metabolism at subcellular resolution.

**Figure 1:**
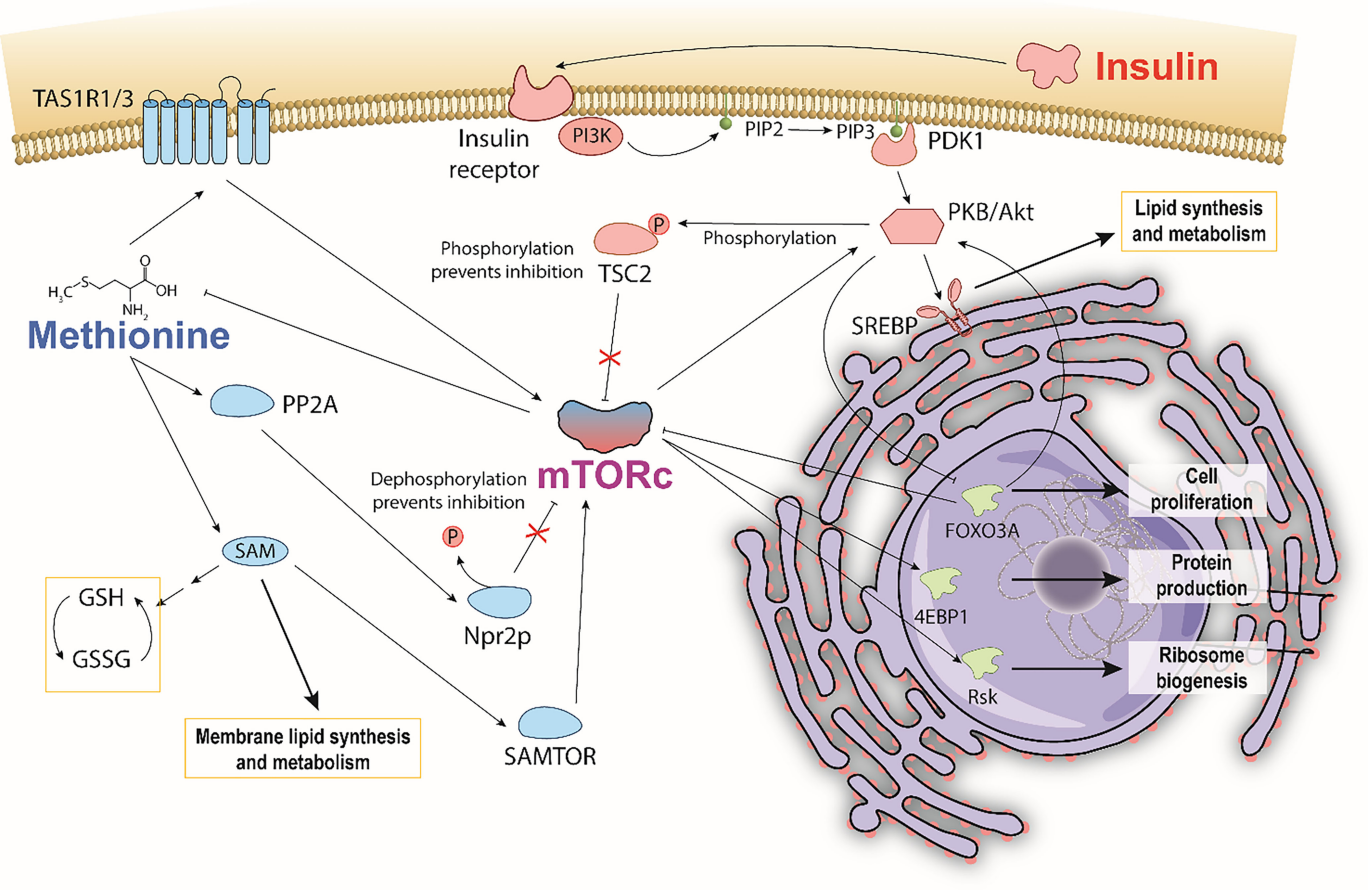
Hypothesized pathway illustrating a potential methionine and insulin interaction mediated through mTOR. Bi-directional control of methionine and mTORc1 depicts general mechanisms by which methionine is sensed by and activates mTORc1. Insulin also activates mTORc1 by phosphorylating TSC2, and consequently affecting mTORc1 regulation of methionine. Insulin stimulates SREBP mediated lipid synthesis and metabolism. Methionine stimulates SAM PC and PE membrane lipid synthesis. Increased production of reduced glutathione *via* SAM is thought to reduce the extent of lipid peroxidation.

Despite the many mysteries of TNBC, a documented hallmark is its hyperactivity of mammalian target of Rapamycin (mTOR) pathways, which play important roles in glucose, protein, and lipid metabolism (19–23). Insulin and L-methionine (an essential amino acid involved in protein translation, genetic/epigenetic control, nutrient sensing, and redox homeostasis) (24) are both involved in mTOR pathways but have not been directly studied in tandem to date (25–30). This is due, in part, to previous studies that observed MDA-MB-231 cells to be insulin insensitive to mitogenic effects, despite having many receptors that bind insulin (31). Other studies observe insulin effects in the same cell line, and there is currently no consensus on the independent effects of insulin. With respect to TNBC, insulin and methionine both independently drive cancer proliferation (32–35) and affect lipid metabolism (25, 34, 36–40), and separate studies indicate insulin metabolism directly affects the uptake of amino acids in yeast and dogs (41, 42). Given the well-documented relationships between insulin, methionine, and mTOR, it is possible that TNBC’s mTOR hyperactivity exhibits a unique lipidomic response to insulin and methionine manipulation. The conceptual pathway detailing macroscopic mTOR-mediated lipid response to insulin and methionine (**Figure 1**) highlights the points discussed in this paper. Lipid peroxidation, *de novo* synthesis, and chemical diversity can all be investigated using optical techniques that provide subcellular spatial and chemical information. Given that TNBC has been an archetype for methionine dependence (35), and that PI3K/AKT/mTOR is a key driver of the aggressive biology of TNBC (23), the interplay between methionine and insulin, coupled with the perspective of lipid biology, may illuminate promising directions for future therapeutic research.

Non-linear optical techniques such as coherent Raman scattering microscopy and two-photon fluorescence (TPF) microscopy have been used to profile breast cancer metabolism by revealing correlations between cancer metastasis and cellular redox state, and lipid metabolism (43). Recent studies have identified several metabolites implicated in tumorigenesis and lipid metabolism in cancer, such as glutamine (44–46) and serine (47) dependence. Raman spectroscopy/microscopy coupled with D_2_O probing allows for direct visualization of metabolic dynamics of a variety of biomolecules including lipids, protein, and DNA in cells, C. elegans, zebrafish, and rodents by highlighting the newly synthesized macromolecules (48). In this study we first employed spontaneous Raman spectroscopy to differentiate molecular signatures within LDs between TNBC and normal cells. Using D_2_O probing and SRS (DO-SRS) imaging we then examined the impacts of methionine and insulin on lipid metabolism in cancer cells. The effects of methionine and insulin on cellular respiration and lipid peroxidation were also examined by using TPF microscopy. To analyze the rich chemometric dataset and inspire targeted image analyses, we applied a relative entropy approach to Raman spectra for the first time. This method can quickly highlight distinct or tandem effects of independent variables in any Raman spectroscopy study.

## Results

### Lipid Droplet Metabolism

**Figure 2:**
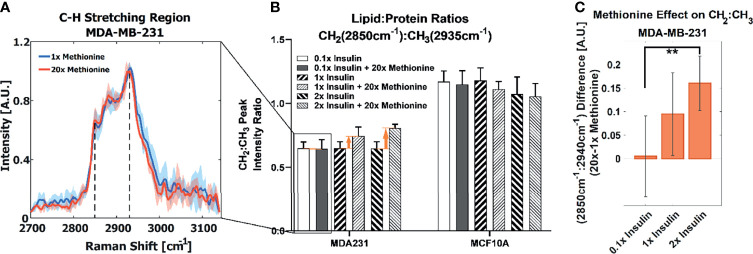
**(A)** Average CH stretching region spectra for TNBC with highlighted CH_2_ and CH_3_ (2850cm^-1^ and 2935cm^-1^) levels ascribed to total lipid and protein content, respectively. Regular and excess methionine groups refer to 0.03g/L and 0.6g/L respectively. Of note, TNBC did not exhibit significant relative lipid and protein changes in the presence of excess methionine. One standard deviation is indicated by shaded areas surrounding the lines. **(B)** With tandem insulin control CH_2_:CH_3_ peak ratios at each insulin and methionine concentration group are shown. 2-way balanced ANOVA results for TNBC cells highlights significance of methionine and insulin-methionine interaction term in lipid:protein ratios. Error bars indicate one standard deviation. **(C)** The difference in CH_2_:CH_3_ ratios for the 15x methionine and 1x methionine groups of the MDA-MB-231 subtype is negligible at the lower insulin concentration but is increased ten-fold in the 2x insulin case. Error bars are propagated in quadrature from **(B)**. **P < 0.01.

We first examined the effects of methionine on LD metabolism in TNBC cells (MDA-MB-231), luminal A breast cancer cells (MCF-7), and normal breast epithelial cells (MCF10A, as a control) by adding excess (20x) methionine to the growth media supplemented with 50% D_2_O. Cells were scanned by using a spontaneous Raman spectroscopy, and revealed that TNBC cells most starkly contrasted MCF10A cells with respect to overall lipid content (CH_2_ stretching at 2850cm^-1^). This attenuated lipid:protein ratio difference between excess and physiological methionine concentrations is shown in **Figure 2A**. This absence of marked differences is also personified by poorer ReLu neural network classification between TNBC cells with and without excess methionine (**Figure S3**). Despite the absence of insulin in TNBC cell culture growth media recipes (49), we then added various concentrations of insulin (1mg/L, 10mg/L, and 20mg/L, correspondingly, 0.1x, 1x and 2x) to the media and evaluated its interaction with methionine in both cell lines. In this second part, insulin concentration in growth media was modulated in tandem with methionine, and augmented effects in several Raman spectral regions were observed, including the C-H stretching region, which illustrates the relative contents of CH_2_ (lipid) and CH_3_ (protein) (**Figure 2B**). TNBC contrasted MCF10A cells which exhibited decreased lipid:protein ratios in the presence of excess methionine at all insulin concentrations. Importantly, it was found that the difference in lipid:protein ratio between excess and physiological methionine increased with the addition of insulin in TNBC. **Figure 2C** highlights this effect, marked by orange arrows in **Figure 2B**, and supports potential interactions between insulin and methionine. A significant interaction term was confirmed by 2-way ANOVA (**Table S1**) in TNBC.

**Figure 3:**
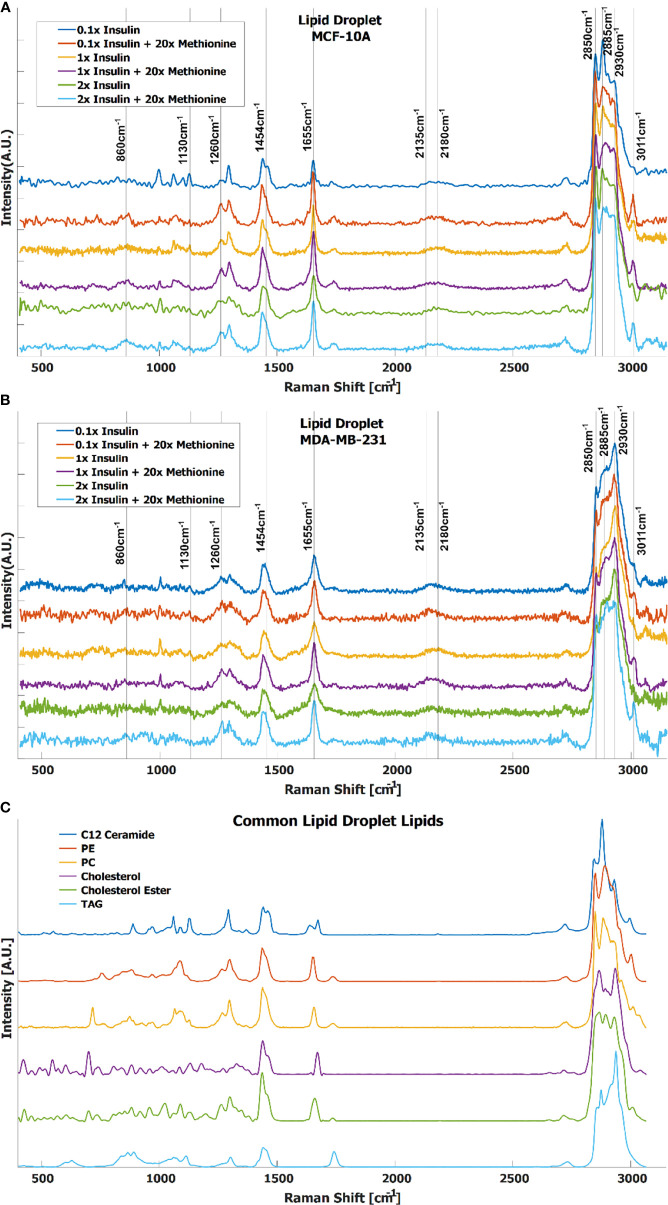
Average spectra of LD from **(A)** MCF-10A and **(B)** MDA-MB-231 cells under various methionine and insulin concentrations. Manual identification of potential Raman peak targets is highlighted with vertical lines and labels. For example, the CD_Lipid_ peak in the cell silent region at 2135cm^-1^ has a noticeable increase relative to the CD_Protein_ peak at 2180cm^-1^ in the excess methionine groups. This could indicate preference for *de novo* lipogenesis in excess methionine environments. **(C)** Raman spectra of common lipid species in LD, in descending order of prevalence.

In TNBC cells, the ratio of total lipid-to-protein did not change with the increase of methionine concentration alone, but slightly decreased in normal cells (**Figure 2B**). With the addition of insulin, this difference was augmented with higher concentrations of insulin (**Figure 2C**). At this point, it is still unclear whether *de novo* lipid synthesis increased alone, or if protein synthesis decreased, or some combination of both. Perhaps *de novo* lipid synthesis decreased, but not as much as protein synthesis. This clarity entails DO-SRS, which provides insight into *de novo* synthesis. As cells incorporate deuterium from heavy water into macromolecules such as lipids and proteins, the C-D bonds in the newly synthesized molecules become visible in the cell silent region around 2150 cm^-1^. Even though lipids and proteins are the main biomolecular constituents of cells, the CH_2_ and CH_3_ peaks may only paint part of the picture. **Figures 3A, B** shows average Raman spectra of both cell lines treated with D_2_O and different concentrations of methionine and insulin. These spectra are consistent with previous LD studies using Raman spectroscopy, which display minute protein peaks in the fingerprint region such as the phenylalanine peak at 1000cm^-1^ and amide I-III peaks at 1660cm^-1^, 1450cm^-1^, and 1200-1300cm^-1^, respectively, as well as elevated CH_2_ stretch at 2850cm^-1^, saturated CH_2_ stretch at 2880cm^-1^ (typical of cholesterol and other saturated lipids) (50), and H-C= stretch at 3010cm^-1^ (typical of unsaturated lipids) (51). Common lipid components of LD are shown in **Figure 3C** in descending order of prevalence. The structure of LDs is such that a phospholipid monolayer surrounds a core of neutral lipids such as cholesterols and TAGs. Less prevalent lipid species such as ceramides, sphingolipids, and their metabolites only account for a small percentage of LD composition, but have gained increasing significance in LD physiology and diseases (52). Furthermore, there are hundreds of apo-lipoproteins on or near the surface of LDs, which may contribute to the observed Raman spectra of LDs. The presence of the C-D peak in the spectra confirm *de novo* synthesis. Some Raman shifts of interest are shown, but minute differences may be difficult to discern by raw visual inspection alone.

**Figure 4:**
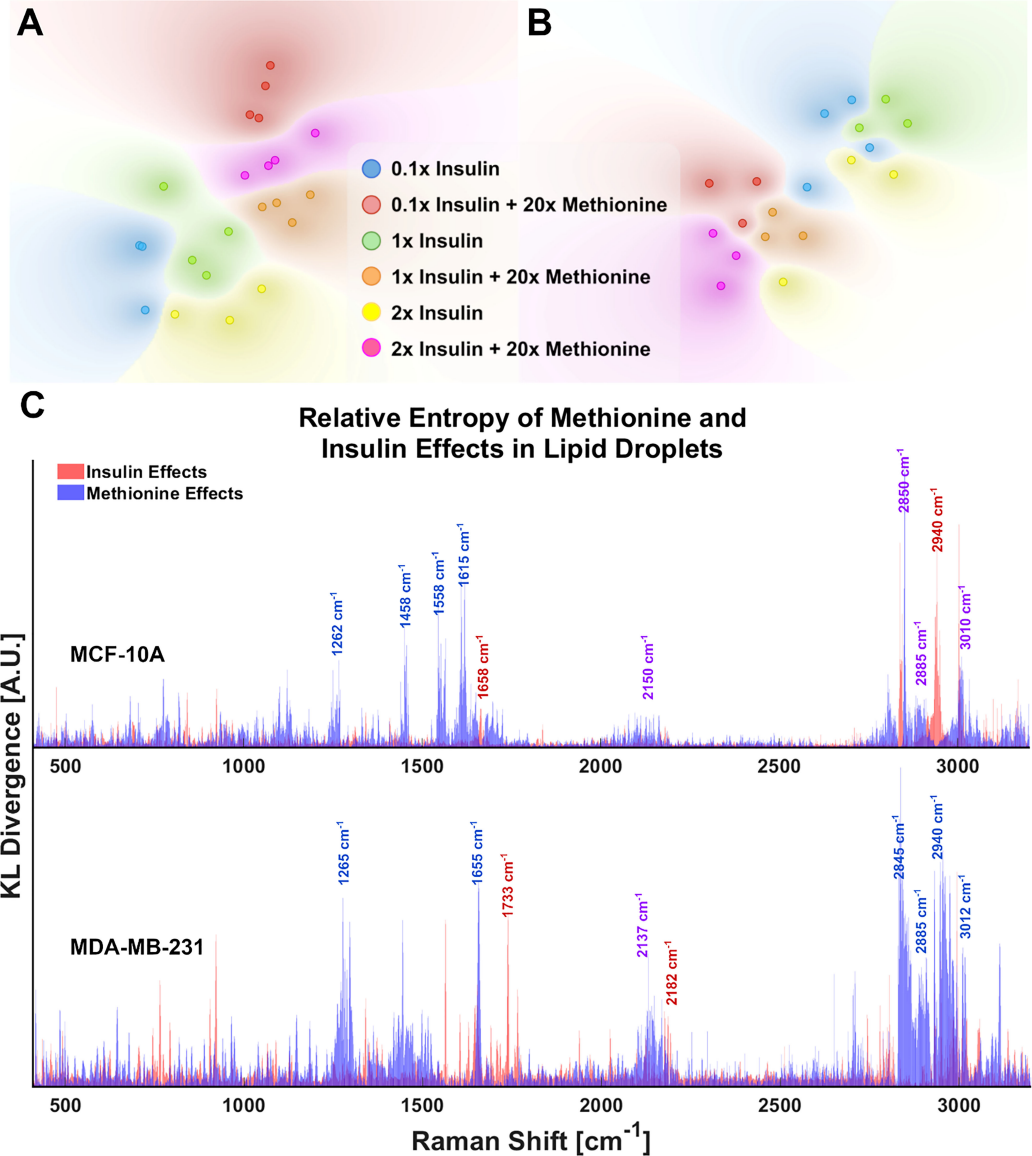
LD chemometric data is sufficient to discriminate cell phenotype **(A)** tSNE separates experimental groups for MCF-10A and **(B)** MDA-MB-231 tSNE plots of the top 10 PCs from PCA of LD spectra. Global structure is preserved, and no exaggeration was applied. Each point represents the average of 5 LD spectra taken from a single cell of the corresponding sample. **(C)** Relative entropy provides a metric for ranking features (Raman peaks) by their ability to classify the spectra as belonging to 20x methionine or 1x methionine groups, as well as 0.1x, 1x, 2x insulin groups. Raman peaks that appear to be influential in both classification schema are denoted in purple text labels for clarity. These cell subtype plots are aligned manually for clarity. Subplots are not generated in such a fashion automatically.

Although the delineated Raman shifts in **Figures 3A, B** highlight several aspects of lipid and protein metabolism, there are others ascribed to lipids and other important molecules as well. Principal component analysis (PCA) shows that 12 principal components (PCs) account for nearly all the variance in the 6 groups of MDA-MB-231 LD spectra. To visualize this while avoiding over-fitting, a t-SNE diagram of the top 10 PCs is shown in **Figures 4A, B**. There exists at least one dimension that discriminates insulin effects and methionine effects on Raman spectra of TNBC LD. Importantly, this demonstrates that LD, alone, contain sufficient chemometric data to discriminate cell phenotypes. This confirms the ability of LDs to reflect cellular state. To date, label-free chemometric demonstrations of this ability are sparse. PCA initialization can be a robust step to reduce dimensions, denoise data, and preserve global structure in t-SNE visualizations, but even though PCA can vectorize these values, the PCs themselves do not take the form of Raman peaks suitable for direct assignment of methionine and insulin effects individually.

Statistical quantification of independent variable effects at every Raman shift entails a new measure in which the separation of insulin and methionine effects, as well as relative significance in class attribution is shown. To quickly rank and visualize all the wavenumber variables that may have been influenced by a particular treatment, the Kullback-Leibler divergence (D_KL),_ a metric for the distance between two distributions for classification problems (53), at each Raman shift is plotted for each metabolite manipulation (**Figure 4C**). This method is also known as relative entropy.

KL divergences of Raman spectra were plotted on the same axes for MCF10A and MDA-MB-231 with selected wavenumbers labeled for clarity (**Figure 4C** **)**. From **Figure 4C**, it is apparent the lipid peak of MCF10A cells at 2850cm^-1^ was heavily influenced by both insulin and methionine concentrations, while the protein peak at 2940cm^-1^ seems to be more heavily influenced by insulin concentration. This contrasts with the MDA-MB-231 TNBC cells in which divergences at most wavenumbers were dominated by the delineation of methionine concentration. Although these representations are not perfect (see **Supplementary Material**), this is especially useful when simultaneous treatment groups have both compound and independent effects. For example, MCF10A spectra (**Figure 4A**) exhibit changes in the unsaturated lipid peak (3010cm^-1^) under either insulin or methionine manipulation, while the TNBC spectra (**Figure 4B**) exhibit changes here (3010cm^-1^) mainly under methionine manipulation. This can be easily seen though the relative entropy at that Raman shift in **Figure 4C**, in which MCF10A has high relative entropy at 3010cm^-1^ when examined along either the insulin or methionine dimension, while TNBC shows a higher relative entropy when examined along the methionine dimension.

**Figure 5:**
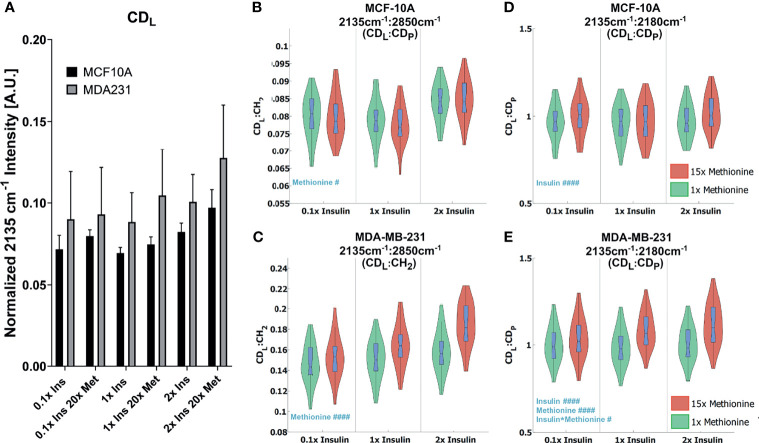
Quantitative *de novo* lipid synthesis **(A)** Normalized CD_L_ intensities show excess methionine stimulates *de novo* lipogenesis. **(B, C)** CD_L_ Ratios show violin box-plots of *de novo* lipid synthesis CD_L_ ratios for MCF10A and MDA-MB-231, respectively. CDL : CH2 illustrates the relative *de novo* lipid synthesized to total ascribable lipid content. Balanced 2-way ANOVA with constrained sum of squares results of CD_L_ ratios shows methionine concentration significantly influenced the CD_L_: CD_P_ ratio in both MCF10A and MDA-MB-231 lipid droplet spectra with rejection levels of ^#^P < 0.05 and ^####^P < 0.0001, respectively. **(D, E)** CD_L_: CD_P_ illustrates the relative *de novo* lipid and protein synthesized biomolecules for MCF10A and MDA-MB-231, respectively. Values were taken from spectra of lipid droplets only. while. There was no significant evidence of interactions between these two independent variables for these ratios. Balanced 2-way ANOVA with constrained sum of squares results of CD_L_ ratios indicate insulin significantly influenced the CD_L_: CH_2_ ratio in MCF-10A lipid droplet spectra with a rejection level of ^####^P < 0.0001, but no significant evidence of interactions between these two independent variables. However, in TNBC insulin, methionine, and the interaction term significantly influenced the CD_L_ : CH_2_ ratio in MDA-MB-231 lipid droplet spectra with a rejection level of ^####^P < 0.0001 and ^#^P < 0.05 for the individual and interaction terms, respectively.

While excess methionine appears to decrease the lipid-to-protein ratio in MCF10A cells and increase the ratio in MDA-MB-231 cells, the results do not necessarily indicate discrepant rates of *de novo* lipogenesis since these values are affected by both synthesis and degradation of lipid and protein. For instance, the decreased lipid-to-protein ratio might be due to enhanced lipid utilization. To explore how much lipid and protein were synthesized, we quantitatively examined the carbon-deuterium (CD) peaks at 2135cm^-1^ (*de novo* synthesized lipids, CD_L_) relative to 2180 cm^-1^ (*de novo* synthesized proteins, CD_P_), and 2850cm^-1^ (total lipids, CH_2_) for each treatment group (48) (**Figures 5A, B**). **Figure 5C** shows that excess methionine stimulates *de novo* lipogenesis in TNBC. Together, **Figure 5** illustrates both direct and relative *de novo* lipid and protein synthesis and metabolism, and informs the potential reasons for the discrepant lipid:protein effects of excess methionine on TNBC and normal-like breast cells.

Two-way ANOVA (**Table S2**) confirms a significant interaction term for methionine and insulin concentrations in TNBC for the *de novo* synthesized lipids relative to the total lipids (**Figures 5A, B** Right). Contrarily, only the insulin independent variable was significant for the MCF10A in the *de novo* synthesized lipids relative to the total lipids (**Figures 5A, B** Right), but no interaction term, or even a significant methionine term. Only in TNBC did the methionine term have a significant impact on this ratio, which lead us to believe the discrepant effects on CH_2_:CH_3_ ratios we observed (**Figure 2B**) might arise from differential *de novo* lipogenesis, rather than protein synthesis and metabolism. Since the excess methionine stimulated *de novo* lipogenesis (**Figure 5C**) and was a significant term in the ratio of *de novo* synthesized lipids to proteins for both cell lines (**Figures 5A, B**), methionine is likely to preferentially stimulate lipid production more than protein production. Despite these findings, the relative proportion of lipids to proteins in MCF10A still decreases under excess methionine (**Figure 2B**). Therefore, either the pool of proteins must be getting larger, or the lipid utilization must increase. Excess methionine did not stimulate lipid utilization because CD_L_ : CH_2_ did not significantly increase (**Figure 5A** Right). This leads us to believe that the protein signal must increase excess methionine. However, excess methionine did not stimulate protein production faster to a greater extent than lipid production since the CD_L_ : CD_P_ slightly increased under excess methionine (**Figure 5A** Left). If there was no relative increase in protein nor decrease in lipids, then MCF10A may not breakdown proteins as much in the presence of excess methionine, or uptake and retain the excess methionine itself more efficiently than TNBC. The hydrophobic amino acid can interact with the acyl chains of the fatty acids in lipid droplets, and since the excess methionine supplied was not deuterated, this protein would not appear in the cell silent region. This way, the excess methionine can affect the CH_3_ peak without affecting the CD_P_ peak, and explain the behavior observed in **Figures 2**, **5**. Excess methionine can also incite endoplasmic reticulum stress due to complex interactions with cysteine pathways since both are sulfur containing and are critical in protein folding due to disulfide bonds. These misfolded proteins may be sequestered by LDs differently across subtypes.

### Morphological Changes in Lipid Droplet

**Figure 6:**
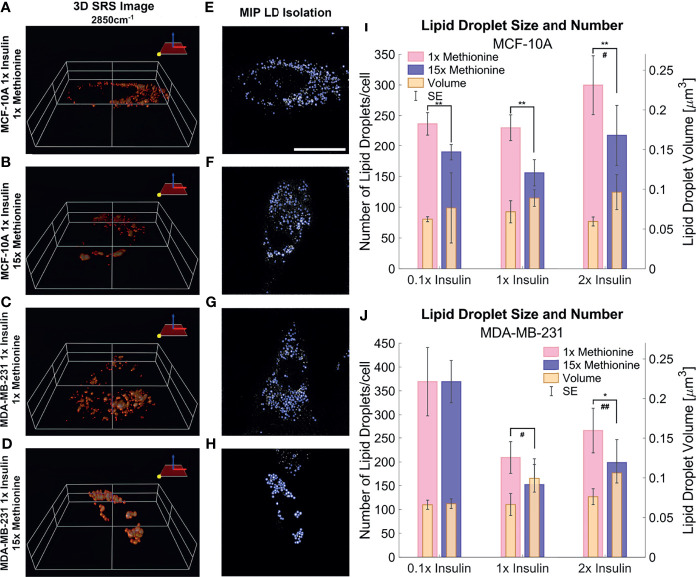
3D SRS image lipid droplet analysis **(A–D)** 3D isosurface reconstructions of the single cell SRS images taken at 2850cm^-1^. **(E–H)** LD segmentation shows representative maximum intensity projections of SRS image stacks shown in **(A–D)** with lipid droplets highlighted in blue outlines. **(I, J)** Quantitative LD structure summary shows average lipid droplet number and volume for each experimental group. Excess methionine groups display decreased lipid droplet number and increased size. Lipid droplets also appear qualitatively more clustered in excess methionine as well. Scale bar is 20 µm. Two-tailed t-tests were performed between each pair of bars to highlight excess methionine effects. Asterisks ‘*’ correspond to the following p-values for LD number: *P < 0.05, **P < 0.01. Octothorps ‘#’ correspond to the following p-values for LD volume: ^#^P < 0.05, ^##^P < 0.01. Scale bar is 20 µm.

3D SRS images were taken for each individual cell at 2850cm^-1^ (**Figures 6A–D**) to assess the size and number of LDs more accurately. LDs were computationally segmented using MATLAB (**Figures 6E–H**) to acquire individual LD volume and number of LDs per cell. The addition of excess methionine produced the most noticeable changes in lipid droplet morphology – a decrease in lipid droplet number but increase in volume. This effect was observed in both MCF10A and TNBC cells (**Figures 6I, J**). Of note is the insulin restricted case in TNBC cells, which had no discernible change to lipid droplet number or size. Qualitatively, the lipid droplets also appeared more clustered in excess methionine cases. Lipid droplet volume was also observed to slightly increase from restricted insulin (0.1x) to physiological and excess insulin (1x and 2x) in TNBC under excess methionine conditions. This corroborates the potential interplay between insulin and methionine in TNBC.

**Figure 7:**
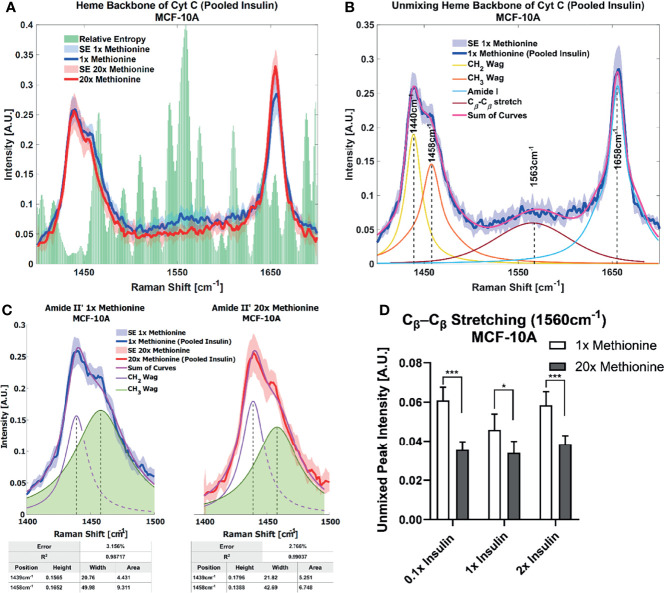
Spontaneous Raman Spectroscopy detects CytC presence and protein folding **(A)** normal vs excess methionine Expanded view of lipid droplet spectra grouped by methionine concentration shows a high relative entropy in the 1550cm^-1^ region, ascribable to the heme backbone of cytochrome C. **(B)** Unmixing Peaks with four peaks using a Gaussian-Lorentzian blend yields an error of 2.367% and an R^2^ of 0.98854. **(C)** Amide II’ Peak Shoulder shows an **e**xpanded view of normal and excess methionine groups’ Amide II’ regions highlight a relatively narrowed shoulder at 1458cm^-1^. Unmixed peaks follow the overall shape of the average Amide II’ peaks, with the error and correlation coefficient reported in the table below. Width and area information is also summarized in the table to clearly communicate the disparate shoulder widths. **(D)** Quantitative summary of the heme backbone unmixed peak intensities for each experimental condition of MCF-10A cultures shows decreased spectral presence. Two-tailed t-tests were performed between each pair of bars to highlight excess methionine effects. Asterisks ‘*’ correspond to the following p-values: *P < 0.05, ***P < 0.001.

**Figure 8:**
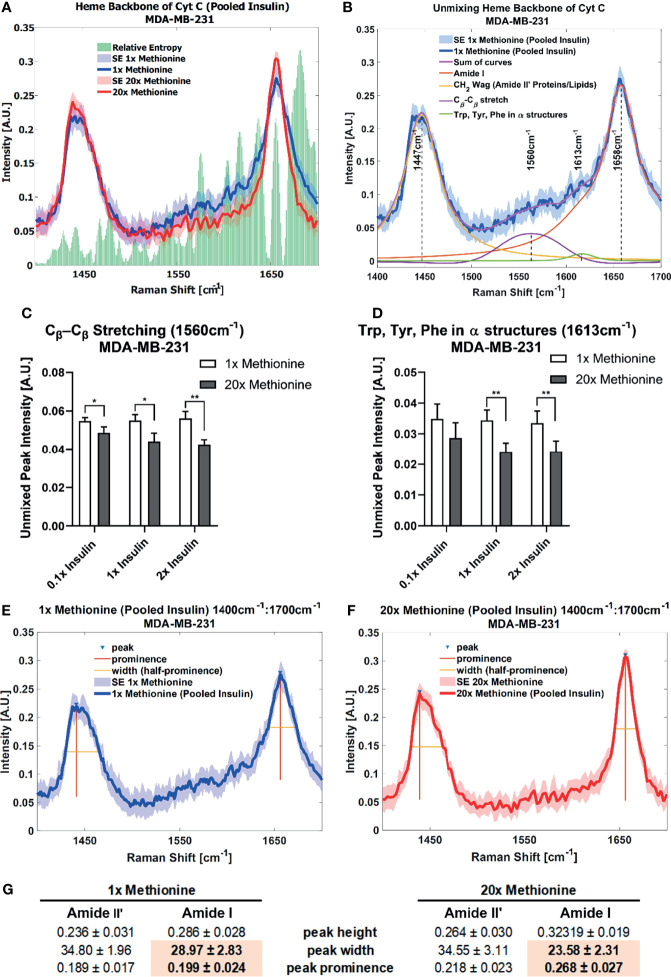
Spontaneous Raman Spectroscopy detects CytC presence and protein folding differences **(A)** normal vs excess methionine shows expanded view of lipid droplet spectra grouped by methionine concentration shows a high relative entropy in the 1550cm^-1^ and 1650cm^-1^ regions, ascribable to the heme backbone of cytochrome C and side chains of tryptophan, tyrosine, and phenylalanine, respectively. **(B)** Unmixing peaks with four peaks using a Gaussian-Lorentzian blend yields an error of 2.620% and an R^2^ of 0.98544. **(C, D)** Quantitative summary of the unmixed peak intensities for each experimental condition of MDA-MB-231 cultures shows decreased spectral presence of cytochrome C. **(E, F)** Amide I peak width shows an expanded view of lipid droplet from 1x Methionine (LEFT), and 20x Methionine (RIGHT) experimental conditions. Two-tailed t-tests were performed between each pair of bars to highlight excess methionine effects. Asterisks ‘*’ correspond to the following p-values: *P < 0.05, **P < 0.01. **(G)** Peak analysis shows that the peak prominence and peak width at half prominence is significantly narrower at the Amide I region in excess methionine lipid droplet spectra.

Lipid droplet distribution can be a major indicator of cell cycle status, nutrient availability, and ER stress (54). LD size may influence the degree to which beta oxidation occurs in cells and be affected by mitochondrial recruitment during LD expansion in nutrient rich environments. Regardless, the physical contact between these organelles is thought to mediate their proper function (55, 56). A label-free approach to identifying mitochondrial presence near lipid droplets may be the spectral presence of cytochrome C (cytC), which is found in the intermembrane space of mitochondria. Some peaks canonically representative of cytC are the heme backbone at 1558cm^-1^ and the side chains of tryptophan, tyrosine, and phenylalanine in alpha structures at 1610cm^-1^ (57) which were weakly present near the fingerprint region of the spectra in MCF10A and TNBC cells. It was found in **Figures 7A**, **8A** that excess methionine cases displayed a diminished spectral presence of unmixed cytC peaks. This suggests LDs in excess methionine may cluster near other organelles such as lysosomes, or even with other LDs for fusion events, instead of co-localizing with mitochondria for energy. Examples of Gaussian-Lorentzian peak unmixing for MCF10A and TNBC (**Figures 7B**, **8B**), respectively, with quantitative summaries in the form of bar graphs (**Figures 7D**, **8C, D**). The number of unmixed peaks was optimized such that the overall fit is accurate, while the unmixed peaks are easily ascribed to canonical protein and lipid deformations. The Amide II’ region contains various CH_2_ and CH_3_ deformations such as wagging, stretching, scissoring, and twisting (58, 59). The Amide I region contains secondary structure information and has been used to study proteins such as collagen (60). Between these peaks lies the C-C bond of the heme backbone. MCF10A and TNBC exhibited distinct peak shapes in all areas of this region. In MCF10A, the Amide II’ peak had a narrower shoulder at 1458cm^-1^ under excess methionine (**Figure 7C**), while TNBC had a narrower Amide I peak under excess methionine (**Figures 8E–G**). The Amide I and II’ regions also contain protein and lipid information and have various assignments in the literature. **Figure 8G** quantifies the width and prominence of the Amide I peak in TNBC with and without excess methionine. Results indicate altered protein folding, in which methionine plays crucial roles. Methionine is not only a protein translational initiator, but its metabolism is also involved in purine synthesis, epigenetic control, and secondary disulfide bond formation (24). Misfolded proteins have tangible effects on ER stress and lipid droplet distribution and chemistry (13), as these proteins have been shown to accumulate in LDs destined for proteasomal breakdown (54). In this manner, LDs may serve as reservoirs and chaperones to mitigate lipid and protein toxicity. Although further investigations are required to confidently assign the phenomenon observed herein, the fact that consistent alterations in these areas were observed using label-free vibrational imaging techniques sets the stage for more in-depth studies of dietary methionine-controlled protein folding in breast cancer cells. **Figure 7C** quantifies the changes in the Amide II’ peak of MCF10A and may indicate altered lipid and protein structure as well. Various bond deformations occur at slightly different wavenumbers, with CH_2_ scissoring being red-shifted with respect to CH_2_ stretching. Acyl chains of different length and saturation may influence the degrees to which each of these deformations take place. Further investigation into purified LD content with other techniques such as gas chromatography and mass spectrometry are warranted. Spectroscopic data are usually sensitive to baseline correction, background subtraction, and normalization methods, and are therefore better suited to relative observations, while chromatography and spectrometry offer absolute quantification and detailed chemical structure. Conjugating these techniques is beyond the scope of this label-free optical platform, but is promising and critical step in progressing this technology.

### Lipid Peroxidation Status

**Figure 9:**
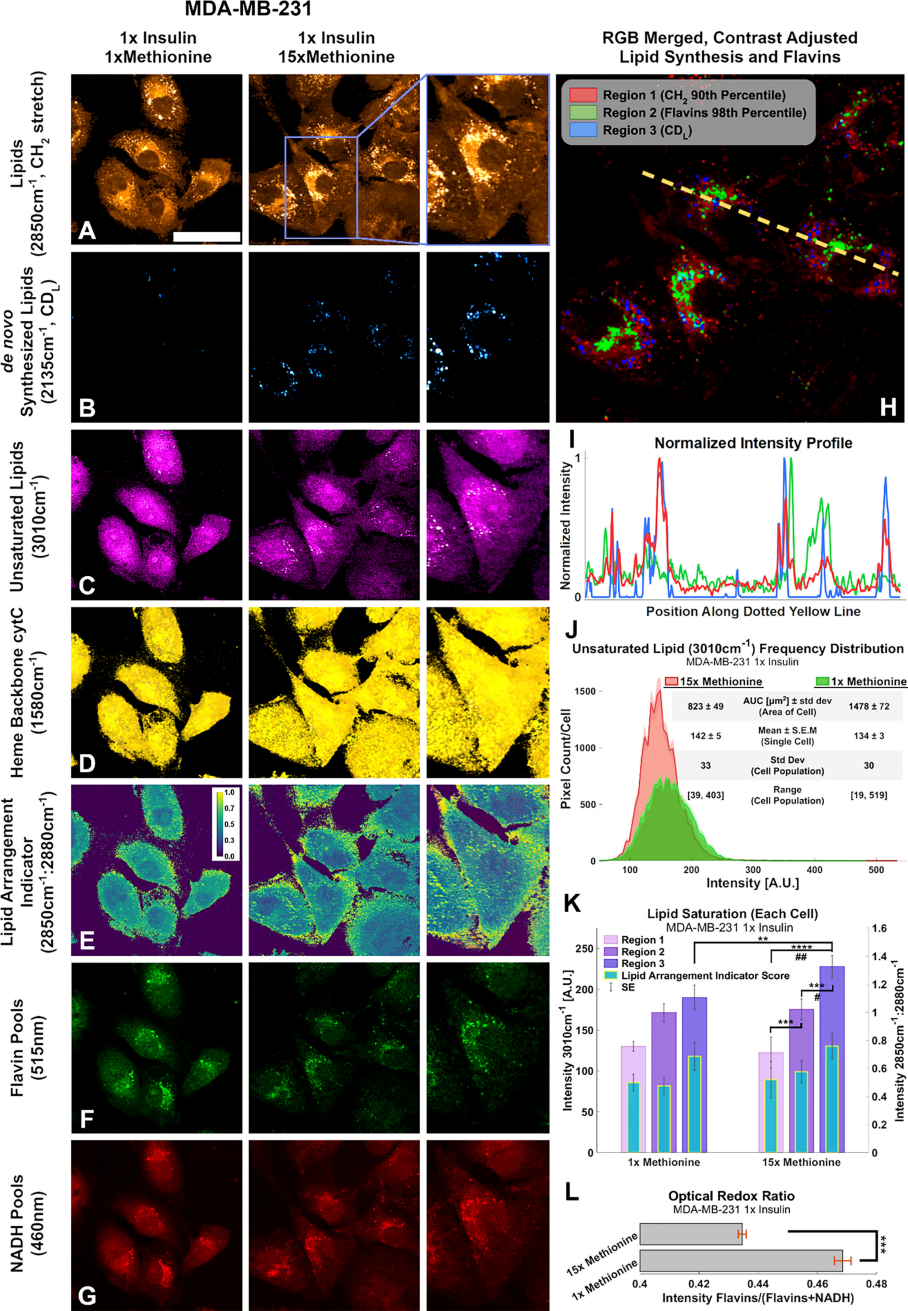
Multi-modal optical analysis depicts MDA-MB-231 1x insulin sample images demonstrate the conjugated SRS and TPF system. **(A-G)** Multichannel Images illustrate SRS and TPF image channels of interest for lipidomic responses to excess methionine. **(H)** Overlaid Composite Regions image of the 15x Methionine lipid (CH_2_), flavins, and *de novo* synthesized lipids (CD_L_). Channels were masked according to the indicated thresholds using ImageJ and contrast was adjusted for optimal clarity. **(I)** Intensity profile plot depicts the intensities of pixels along the dotted yellow line shown in **(H)** of each of the three composite channels. **(J)** Composite intensity histograms of the unsaturated lipid channel (3010cm^-1^). Bolded distribution outlines represent the average frequency of pixel intensities among the cells in each group. Shaded areas around the bolded distribution outline represent the standard error of the mean of each bin of pixel intensities. Each distribution curve represents the pixel intensities of a cell sampled from the experimental condition. **(K)** Quantitative Lipid saturation summary depicts the 3010cm^-1^ pixel intensities (Left axis) in each of the three regions shown in **(H)** of a typical cell from the indicated experimental condition. Additionally, the lipid arrangement indicator ratio (2850cm^-1^:2880cm^-1^) for each of the regions in **(H)** is also depicted for the typical cell from each experimental condition. Two-tailed t-tests were performed between each pair of bars to highlight excess methionine effects. Asterisks ‘*’ correspond to the following p-values for the unsaturated lipid peak (3010cm^-1^) intensities: **P < 0.01, ***P < 0.001, ****P < 0.0001. Octothorps ‘#’ correspond to the following p-values for the Lipid Arrangement Indicator (2850cm^-1^: 2880cm^-1^) intensities: ^#^P < 0.05, ^##^P<0.01. **(L)** Optical redox ratio (Flavin/(Flavin+NADH) autofluorescence intensity) for the typical cell from each experimental condition. Results corroborate and extend spectral data findings, as well as previous third-party studies. Scale bar is 50um. Two-tailed t-tests were performed between each pair of bars to highlight excess methionine effects. Asterisks ‘*’ correspond to the following p-values for the unsaturated lipid peak (3010cm^-1^) intensities: ***P < 0.001.

Another global lipid response to excess methionine takes form in the lipid peroxidation status. Under oxidative stress, long chain unsaturated fatty acids can undergo a vicious cycle of lipid peroxidation (51). Several Raman shifts have been used to describe the degree of unsaturation of fatty acids, including the one near 3010 cm^-1^ that corresponds to the H-C= stretching region (51). Interrogating the relative entropy plot in **Figure 4C**, we find that the saturated lipid peak at 3010 cm^-1^ and the lipid peak at 2850 cm^-1^ both rank highly for both cell types, but TNBC is more heavily influenced by methionine concentration. That is, we can see from the spectroscopic data that MCF10A, whether L-methionine was normal or in excess, expressed relatively different levels of unsaturated lipids depending on the level of insulin. This suggests that *de novo* synthesis of branched chain fatty acids, or perhaps their accumulation in LDs was upregulated in excess insulin conditions. So, while insulin was critical in influencing *de novo* synthesis of lipids in TNBC, it may not influence lipid peroxidation as much as methionine does. **Figure 9** shows the effects of excess methionine in TNBC using multi-modal optical techniques.

Conjugated SRS and TPF microscopy display spatial distributions of points of interest regarding excess methionine effects in TNBC cells (**Figures 9A–G**). These results also corroborate with the spectroscopic data. **Figure 9B** reveals that the cells undergo enhanced *de novo* lipogenesis under excess methionine with respect to control groups. Contrary to expectations, the unsaturated lipid signal in the excess methionine group was weaker than the control overall but was stronger near the large lipid droplets (**Figure 9C**). This information is lost in spectral acquisitions alone because spectra were obtained from lipid droplets only. Excess methionine treated cells exhibited larger cross-sectional area (**Figure 9J**) and may be due to the cells being more spread out and flatter. Due to the point spread function of the confocal laser scanning microscope, this spreading out of the cells may contribute to an apparent decrease in concentration of unsaturated fatty acids because the scattering cross section along the beam path is smaller. Consistent contrast makes it difficult to discern the abundance of smaller lipid droplets in the control images without oversaturating the excess methionine images. There were no discernible differences in spatial distribution of heme groups at the 1580 cm^-1^ (**Figure 9D**), but co-localization algorithms may help in future studies. The indicator of crystalline arrangement in lipids corresponding to the symmetric:antisymmetric CH_2_ stretching ratio indicates that the excess methionine group may have less lipid saturation near the plasma membrane (**Figure 9E**). Higher ratios would indicate a lower concentration of 2880 cm^-1^ species, which has been ascribed to the Fermi resonance of CH methylene groups (50). This ratio has been found to inversely correlate with thermodynamic stability, and when the ratio is larger, there may be less lattice order in the structure (61). In the context of cell membranes, fluidity and saturation are critical functional properties, and the decreased lipid saturation score near the plasma membrane may also contribute to the observed “flattness” of the cells with excess methionine, as the cells may be able to spread out more easily.

Different areas of the cells provide niche microenvironments, in which lipid peroxidation may vary. Three subcellular regions of interest include where all lipids exist (**Figure 9H**, region 1), where flavins are more present (region 2), and where newly synthesized lipids are present (region 3). As shown in **Figures 9H, I**, these regions do not necessarily overlap. Flavins have been shown to report on oxidative stress, and certain flavin enzymes have been associated with lipid peroxidation as well. The quantitative image analyses of unsaturated lipids (3010cm^-1^) and the lipid arrangement indicator ratio (2850cm^-1^:2880cm^-1^) are summarized in **Figure 9K**, in which distinct regions are separately quantified. **Figure 9J** highlights a larger cross-sectional area of the imaged cells, which may be afforded by a more fluid cell membrane. In certain cells, oxidative stress has been found to increase lipid saturation for protection. Furthermore, the presence of higher ratios near LDs suggests there is less synthesis of saturated lipid species as well. Finally, flavin autofluorescence decreased in the presence of excess methionine (**Figure 9F**), while NADH autofluorescence remained more consistent (**Figure 9G**). The flavin/(NADH + flavin) ratio has been shown to be an indicator of oxidative stress and estimator for NAD^+^:NADH (62). Results corroborate with previous studies in which this ratio was used to differentiate breast cancer cell lines (63), with the TNBC having relatively weaker flavin autofluorescence than the normal-like cell type. Under oxidative stress, this ratio has been shown to increase. A decrease here (**Figure 9L**) may demonstrate the antioxidant properties of methionine. Flavin autofluorescence data is summarized in **Figure S2**.

## Discussion

For the first time, the unique lipid metabolism of triple negative breast cancer was studied under tandem excess methionine and insulin conditions, and revealed key insights that span the metabolic, spatial, and biochemical dimensions. Not only did this study confirm lipid droplets are reflective of cellular phenotypes and demonstrate their efficacy in classifying breast cancer subtypes, and even phenotypes, it improves morphological analysis using 3D imaging, as opposed to 2D, and efficiently displays relevant chemical disparities using the first demonstration of relative entropy for Raman data. Considering the critical impact lipid metabolism has on the progression of diseases such as cancer, the analyses on lipid saturation and peroxidation, optical redox status, and LD size and distribution solidify the effects of methionine and insulin, which may prove to be therapeutic targets for breast cancer in the future.

These experiments demonstrate the power of nearly label-free optical techniques to probe LD phenotypes for the study of TNBC’s unique metabolism. Methionine dependence, also known as the Hoffman effect, has been explored in TNBC and other cancers, but fewer studies explored the effects of excess methionine, and fewer still, the tandem manipulation of methionine and insulin. Upon the addition of insulin in TNBC growth media, macromolecular changes appeared in the CH stretching region of excess methionine treated cells, as the CH_2_:CH_3_ ratio increased in TNBC, but decreased in MCF10A control cells. A potential pathway that involves both insulin and methionine in LD metabolism may be mediated by TNBC’s elevated mTOR activity, and was explored through the chemometric, spatial, and molecular imaging dimensions with subcellular resolution. Currently the stoichiometric mass action of this pathway remains to be investigated in these breast cancer subtypes, but several studies have linked methionine, mTOR, and insulin signaling pathways (25–30), albeit transitively. Paramount in this investigation is the implication of these metabolites in the pursuit of TNBC diagnosis and treatment. Unmixing the interplay between insulin and methionine may afford targeted therapies that address the rampant lipid metabolism that facilitates breast cancer progression.

LD chemical composition also demonstrated excellent classification ability, as lipid and protein Raman cross sections are not only larger, but also very diverse and highly implicated in metabolic cellular states. Classification of breast cancer subtypes, and even more so their phenotypic states, can be critical in improving patient outcomes due to the necessity of early diagnosis. MCF10A cells may exhibit differential protein metabolism by retaining scavenged methionine or not metabolizing proteins as much as TNBC, which is due, in part, to very different doubling times. Several other considerations including LD size may also contribute to these disparities, as larger LDs have a greater volume:surface area ratio, and thus a greater lipid:protein ratio since the apolipoproteins stud the phospholipid surface of the lipid core. LD fusion can affect this as well, since newly synthesized LDs may have a diluted CD signal if fused with older LDs. Further investigation is warranted to uncover the details of differential lipid metabolism in breast cancer subtypes using LDs, but this platform has set the stage for macroscopic observations using efficient optical techniques.

Both TNBC and MCF10A cells exhibited similar spatial information of LDs under these treatments as excess methionine conditions generally decreased the number of lipid droplets while increasing their volume in both cell types, while insulin generally increased both size and number of LDs. Insulin restriction appeared to increase LD number as well, and in TNBC, appeared to attenuate the effects of excess methionine on LD number. This interesting pattern not only suggests that TNBC has unique insulin-mediated lipid metabolism, but that insulin and methionine may have more complex concentration-dependent interactions in general as well. LD distribution also seemed to be more clustered in the excess methionine cases, and we intuit from the diminished spectral presence of cytC that these lipid droplets were less co-localized with mitochondria. Finally, the antioxidant properties of methionine expectedly diminished flavin autofluorescence and resulting lipid droplet spectra showed higher degrees of lipid unsaturation. In **Figures 9K**, L the optical redox ratios and the lipid arrangement indicator ratios indicate that methionine plays a large role in lipid peroxidation and saturation. The degree of saturation of lipids is a critical consideration for cell membrane fluidity, especially in aggressive cancers that can alter their extra cellular matrix (ECM), or those that metastasize and migrate rapidly. For the first time, the dynamics of lipid saturation and peroxidation under nutritional control has been imaged with label-free subcellular resolution.

To broaden the scope of the investigation and capitalize on the rich chemical data of the Raman spectrum, relative entropy was used to rank the features that exhibited the greatest variance between different groups. As expected, there are several areas other than the CH stretching region that offer strong classification ability despite lower Raman intensities. This may be attributed to the higher intensity deviations at higher intensities typical of multiplicative scattering effects. Additionally, the effects of individual nutritional manipulations become clearer with all Raman peaks being visible simultaneously. From this insight, the interrogation of pathways with Raman spectra can be done more efficiently, as the relative entropy scores for each Raman shift can be seen at once, reducing the number of spectra and subplots that need to be displayed. With this demonstration of efficacy, more critical quantitative analyses, as well as algorithmic improvements will be conducted. For example, incorporation of directional shifts in intensities can be made visible on the relative entropy plot, as opposed to absolute distance metrics alone. This will not only identify discriminating variables but will also circumvent the need to manually determine significant ratios, ratio differences, and other trends as well. Further, this relative entropy plot may be useful in feature reduction, so that fewer hyperspectral images may be required for discriminating LD microenvironments and subpopulations. Other methods more directly identify the wavenumbers that contribute the most to a spectrum’s classification, such as the hybrid variable combination population analysis (VCPA) and iterative retaining important variables (IRIV) approach (64). However, due to the large number of variables, IRIV can be time and resource intensive.

The diverse pathophysiology of breast cancer may have important mechanisms involving methionine and insulin that can be studied with optical techniques such as spontaneous Raman spectroscopy and SRS/TPF microscopy. This study also emphasizes that LDs are organelles diverse in structure and function and can yield rich metabolic information when interrogated by Raman techniques. Future studies that involve automated high-throughput acquisitions of spectra and images at more finely tuned concentrations of insulin and methionine may increase the power of the results discussed here. Different distribution fits for the relative entropy algorithm, displaying directionality of peak intensity changes, as well as the multiplexing of dietary manipulations such as glucose, pyruvate, and glutamine may paint a clearer picture of the metabolic dynamics in breast cancer (65, 66). This will also help make hyperspectral imaging more efficient in terms of disk space and clustering ability. Utilizing morphological characteristics and intensity changes to augment classification has not been performed in this study but will be a prudent next step in developing these optical techniques for classification purposes. Additionally, spatial distribution of LD by size and chemometric composition, as well as quantitative descriptions of LD distribution and co-localization would further enrich this investigation. This kind of quantitative hyperspectral image data will bolster the utility of LD analysis in the study of breast cancer, and ultimately improve not only our understanding of the complex disease, but patient outcomes in eventual translation as well.

## Material and Methods

### Experimental Design

**Figure 10:**
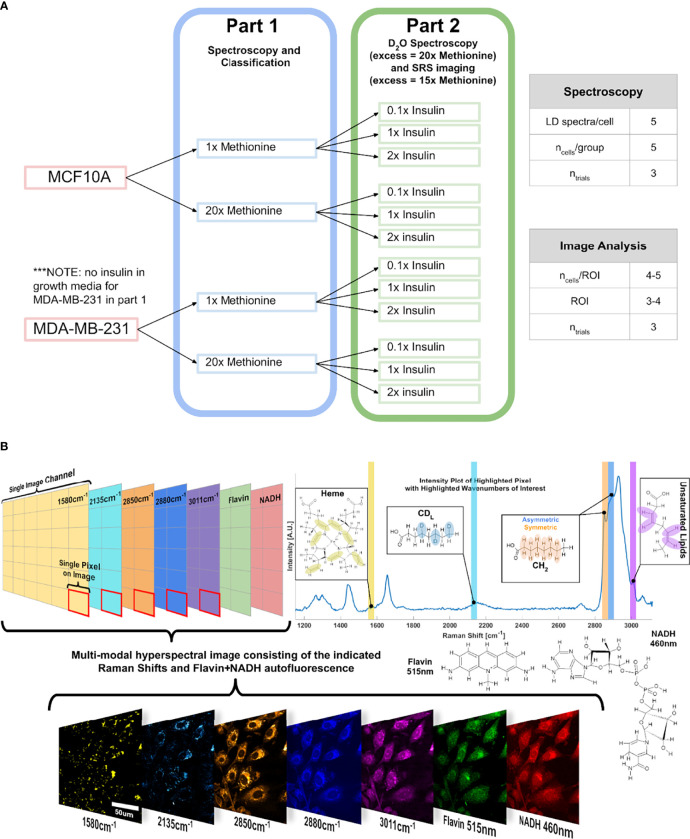
Experimental Design and Points of Interest **A** Experimental design illustration of the groups in this study. Only methionine concentration is modulated at first, and then insulin and methionine were modulated in tandem. DMEM used in this experiment already contains 0.03g/L of L-methionine, which corresponds to the 1x methionine group. NOTE: in part 2, the excess methionine concentration is 20x for the Raman spectroscopy, and 15x for SRS imaging. **(B) **Points of interest where SRS images relating to lipid metabolism are acquired, related to the lipidomic investigation. Hyperspectral Image (HSI) format is shown conceptually to convey the multi-modal approach to quantitative optical analysis. Vibrational modes are color coded, with an example image of a HSI of MCF10A control cells.

An experimental outline is shown below (**Figure 10** **).** First, three cell subtypes were grown in media with either 1x methionine (0.03g/L) or 20x methionine (0.6g/L). Then the experiment was repeated with the addition of 3 insulin concentrations for each of the groups to investigate their relationship.

### Cell Culture

Human triple negative breast cancer cell line (MDA-MB-231) and normal-like breast epithelial cell line (MCF10A) were cultured in Dulbecco’s modified Eagles’ medium (DMEM), supplemented with 5% fetal bovine serum (FBS) and 1% penicillin/streptomycin (Fisher Scientific, Waltham, MA), and incubated with 5% CO_2_ at 37°C. Cell cycles were synchronized using double thymidine block (67). After passaging at 80% confluence, cells were seeded at a concentration of 2×10^5^/mL atop 70% ethanol-soaked cover glass in 24-well plates and incubated for 8 hours. Then the growth media was changed to 50% heavy water (D_2_O) and treatment media as follows.

For MDA-MB-231 and MCF10A cell culture media, 57 mg/L and 42 mg/L L-methionine (M8439, Sigma Aldrich) was added to DMEM for the excess methionine group for spontaneous Raman spectroscopy and SRS imaging (20x and 15x concentration), respectively. The DMEM powder used in this study already contains 0.03g/L (1x concentration) L-methionine and corresponds to the physiological concentration group. The reason for lowering the excess methionine concentration for SRS imaging analysis is because the cell morphological changes were more varied and poorer with 20x methionine, making it more difficult to acquire quantitative metabolic activity from images on a per-cell basis. Insulin (Sigma Aldrich, St. Louis, MO) was added at 1µg/mL, 10 µg/mL, and 20 µg/mL for the 0.1x, 1x, and 2x insulin groups, respectively.

Cells were incubated for 48 hours, which corresponds to a deuterium-retarded cell cycle. Cyclin dependent kinase 1 (CDK1) inhibitor (RO-3066, Sigma) was added with 8 hours remaining to arrest growth before mitosis. Cells were gently rinsed with 1x PBS with Calcium and Magnesium ions at 4°C (Fisher Scientific, 14040216), and finally fixed in 4% methanol-free PFA solution (VWR, 15713-S) for 15 minutes. The cover glass was mounted on 1mm thick glass microscope slides with 120 µm spacers filled with 1x PBS without calcium and magnesium ions. These samples are stored at 4°C submerged in PBS when not in use.

### Spontaneous Raman Spectroscopy

Spontaneous Raman scattering spectra were obtained by a confocal Raman microscope (XploRA PLUS, Horiba) equipped with a 532 nm diode laser source and 1800 lines/mm grating. The acquisition time is 30 s with an accumulation of 4. The excitation power is ~40 mW after passing through a 100x objective (MPLN100X, Olympus). The background spectra were taken for each LD at the same focus plane as the LD and were subtracted from each LD spectrum immediately. Spectra were preprocessed using vector normalization and simplex normalized. Peaks were normalized to the protein peak at 2940 cm^-1^. Previous studies suggest Raman microspectroscopy can quantify lipids non-invasively (68).

### Stimulated Raman Scattering Imaging Microscopy

An upright laser-scanning microscope (DIY multiphoton, Olympus) with a 25x water objective (XLPLN, WMP2, 1.05 NA, Olympus) was applied for near-IR throughput. Synchronized pulsed pump beam (tunable 720–990 nm wavelength, 5–6 ps pulse width, and 80 MHz repetition rate) and Stokes (wavelength at 1032nm, 6 ps pulse width, and 80MHz repetition rate) were supplied by a picoEmerald system (Applied Physics & Electronics) and coupled into the microscope. The pump and Stokes beams were collected in transmission by a high NA oil condenser (1.4 NA). A high O.D. shortpass filter (950nm, Thorlabs) was used that would completely block the Stokes beam and transmit the pump beam only onto a Si photodiode for detecting the stimulated Raman loss signal. The output current from the photodiode was terminated, filtered, and demodulated in X with a zero phaseshift by a lock-in amplifier (HF2LI, Zurich Instruments) at 20MHz. The demodulated signal was fed into the FV3000 software module FV-OSR (Olympus) to form the image during laser scanning. All 3D lipid droplet images were obtained with a pixel dwell time 40 µs with 3-frame averaging for a total imaging speed of ~10-15 min per image stack. Laser power incident on the sample is approximately 40mW.

### Two Photon Fluorescence Microscopy

Autofluorescence of flavins was excited at 820 nm and autofluorescence of NADH was excited at 780nm using the same tunable picosecond laser described in section 2.3. Epi-detected emission of flavin autofluorescence was collected using a 460 nm filter cube (OCT-ET460/50M32, Olympus), and NADH was collected using a 515nm filter. These images were also 512x512 pixels and were acquired with a 12.5 µs/pixel dwell time using a 300mW power at the laser shutter. Autofluorescence images were background subtracted using a rolling ball algorithm with a radius of 50px, which is intended to approximate cell size in these images.

### Data Analysis

#### Spectral Clustering

Previous studies have shown these breast cancer subtypes have unique Raman features (16, 69). Machine learning was conducted to determine the extent to which these features can be used to segregate these subtypes and be augmented using the metabolic dimension of excess methionine. Neural network classification was done using a simple multi-layer perceptron (MLP) model with 100 neurons in the hidden layer and a rectified linear unit (ReLU) activation function for each neuron. An L2 regularization term with hyperparameter α=0.0002 penalizes the model for incorrect classification during learning with cross-entropy loss minimization. The classification is stochastically optimized using an adaptive moment estimation algorithm called Adam. Advantages of this choice of activation function and solver in an MLP include invariance to rescaling gradients, the ability to learn non-linear models, and a natural simulated annealing to optimize the gradient (70). MLP are, however, sensitive to parameter tuning, and all spectra were normalized to have the same range.

The input for the MLP model consists of a matrix of Raman spectra, and a vector of target classes. In this study, target classes are of categorical type and correspond to the cell subtype and metabolite concentration groups. Each spectrum X_i_ is represented as a vector containing m wavenumbers which are each input into a first layer of neurons. Each neuron in the hidden layer accepts the weighted linear combination of input features and applies the ReLU activation function, outputting the data to the output layer. Softmax is used to probabilistically determine the target class of the multiclass model. The model is trained *via* backpropagation to minimize cross-entropy loss with a maximum of 200 iterations in which subsequent weight vectors reflect a subtracted loss gradient according to equation 1 below.


(1) $$W^{i+1}=W^{i}-\in \nabla Loss_{W}^{i}$$

where, ϵ corresponds to the learning rate. A python implementation of model is readily available from scikit-learn v0.24.1 (71). The width of the hidden layer, k=100, as well as its depth of 1 single layer, are tunable depending on the dataset. Larger datasets may require more neurons and deeper networks to perform better. The geometric mean of input variables and classes roughly totaled 100, and an underlying assumption of a simple binary effect of excess methionine and very distinct Raman spectra was comfortable with only a single layer. However, multiplexing of variables such as cell subtype, methionine concentration, and other manipulations may intuitively justify additional hidden layers in future experiments. Classification in this study mainly attempts to highlight the higher dimensionality of methionine’s non-linear effects on breast cancer subtypes, and discuss potential pathway interactions for further investigation. That is, if all breast cancer subtypes responded similarly and to a similar degree, more complex neural nets may not be necessary to achieve good performance.

Principal component analysis (PCA) is performed using Orange 3.26 on the pre-processed data. The first 10 PCs are used as the vectors for tSNE visualization without any exaggeration and a perplexity of 30. A graphical method outline can be found in **Figure S2**.

### Selected Raman Feature Analysis

Spectroscopic data is extracted using MATLAB and is plotted using either MATLAB or Prism 7. To visualize the influence of all Raman peaks on classification simultaneously, relative entropy is employed. For binary classification systems, the amount of data lost in classifying data B as data A is described by the one-dimensional cross entropy equation 2 below.


(2) $$H(A,B)=-\Sigma_{i}^{n}(p_{A}{〚(v〛}_{i})\log p_{B}(v_{\text{i}}))$$

*H*(A,B) is the cross-entropy, *p*_A_(*v*_i_) and *p*_A_(*v*_i_) are probability vectors from the distributions of intensity values a wavenumber variable *v*_i_. Probability vectors can be derived from various distributions, but only gaussian normal distributions were used in this study. The D_KL_ is related to cross-entropy as it is the additional entropy beyond the entropy of the data A. Since both distributions are already labeled and we are not interested in generating probability vectors, but rather supply them, the D_KL_ is described by Equation 3 below.


(3) $$D_{KL}(\text{A}||\text{B})=\sum_{i}^{n}\left(P_{A}(v_{i}\right)\log\left(\frac{p_{\text{A}}(v_{\text{i}})}{p_{\text{B}}(v_{\text{i}})}\right)$$

This divergence is made symmetric by equation 4 below. For this analysis probability vectors are of length 10^7^


(4) $$D_{KL}=\frac{D_{KL}(\text{A}||\text{B})+D_{KL}(\text{B}||\text{A})}{2}$$

For multiclass situations in which the number of groups exceeds two, the average divergence is calculated following equation 5 (72, 73).


(5) $$D(P_{1}\ldots P_{k})=\frac{1}{k(k-1)}\sum_{i}^{k}\sum_{j}^{k}D_{KL}(P_{i}||P_{j})$$

Relative Entropy is an excellent method for quantifying the relative importance of a wavenumber in the discrimination of spectral datasets because it is computationally fast and capitalizes on the tremendous chemometric potential afforded by the spectral resolution of modern spontaneous Raman systems. To date, this method has not yet been employed and reported in Raman spectroscopy applications. Second derivative barcode analysis may be coupled with this method, and improve it, as barcode analysis contains both amplitude and width information (74).

### Image Analysis

Images were processed using MATLAB and ImageJ. 3D image stacks of lipid droplets underwent bandpass filtering to suppress horizontal noise artifacts from laser beam scanning, and smoothed. Lipid droplets received a sphericity score based on Euclidean distance from perfect spheres emanating from the center of mass of the lipid droplet to the surface of the lipid droplet. Those with low sphericity scores were discarded. Autofluorescence images underwent sliding paraboloid background subtraction before manual cell segmentation and measurement was conducted *via* ImageJ.

### Statistical Analysis

All experiments were run in triplicate. LD spectra for display, ratiometric peak analysis, subtype clustering, and relative entropy comprise 5 LD spectra per cell, and 5 cells per experimental group per trial. SRS and TPF images used in multi-modal analysis consist of 3 or 4 ROI of approximately 5 cells per ROI in each experimental group per trial. SRS images used in 3D spatial analysis consist of 4 cells per experimental group per trial. 2-way balanced ANOVA results were consistent between trials, so to communicate the impact patterns of methionine and insulin, results were pooled such that each datum represents a measurement, not a trial mean.

## Data Availability Statement

The original contributions presented in the study are included in the article/**Supplementary Material**. Further inquiries can be directed to the corresponding author.

## Author Contributions

AF and LS conceived the idea and designed the study. AF conducted the experiments, analyzed the data with the input from KH, DC, HZ, WZ and LS; AF and LS wrote and revised the manuscript with the input from all other authors. All authors contributed to the article and approved the submitted version.

## Funding

This work is partially supported by UCSD Startup funds, U54 pilot grant 2U54CA132378-11A1, and Hellman Fellow Award.

## Conflict of Interest

The authors declare that the research was conducted in the absence of any commercial or financial relationships that could be construed as a potential conflict of interest.

## Publisher’s Note

All claims expressed in this article are solely those of the authors and do not necessarily represent those of their affiliated organizations, or those of the publisher, the editors and the reviewers. Any product that may be evaluated in this article, or claim that may be made by its manufacturer, is not guaranteed or endorsed by the publisher.

## References

[1] DeSantis CE Ma J Gaudet MM Newman LA Miller KD Sauer AG. Breast Cancer Statistics, 2019. CA: A Cancer J Clin (2019) 69:438–51. doi: 10.3322/caac.21583 31577379

[2] Dai X Li T Bai Z Yang Y Liu X Zhan J. Breast Cancer Intrinsic Subtype Classification, Clinical Use and Future Trends. Am J Cancer Res (2015) 5:2929–43. PMC4656721 26693050

[3] Olzmann JA Carvalho P. Dynamics and Functions of Lipid Droplets. Nat Rev Mol Cell Biol (2019) 20:137–55. doi: 10.1038/s41580-018-0085-z PMC6746329 30523332

[4] Cruz ALS de Barreto QAE Fazolini NPB Viola JPB Bozza PT. Lipid Droplets: Platforms With Multiple Functions in Cancer Hallmarks. Cell Death Dis (2020) 11:1–16. doi: 10.1038/s41419-020-2297-3 32029741 PMC7005265

[5] Daniëls VW Smans K Royaux I Chypre M Swinnen JV Zaidi N. Cancer Cells Differentially Activate and Thrive on *De Novo* Lipid Synthesis Pathways in a Low-Lipid Environment. PloS One (2014) 9:e106913. doi: 10.1371/journal.pone.0106913 25215509 PMC4162556

[6] Franco D Trusso S Fazio E Allegra A Musolino C Speciale A. Raman Spectroscopy Differentiates Between Sensitive and Resistant Multiple Myeloma Cell Lines. Spectrochim Acta Part A: Mol Biomol Spectros (2017) 187:15–22. doi: 10.1016/j.saa.2017.06.020 28645097

[7] Butler LM Mah CY Machiels J Vincent AD Irani S Mutuku S. Lipidomic Profiling of Clinical Prostate Cancer Reveals Targetable Alterations in Membrane Lipid Composition. bioRxiv (2020). doi: 10.1101/2020.10.27.356634 34362796

[8] Dawaliby R Trubbia C Delporte C Noyon C Ruysschaert J-M Van Antwerpen P. Phosphatidylethanolamine Is a Key Regulator of Membrane Fluidity in Eukaryotic Cells. J Biol Chem (2016) 291:3658–67. doi: 10.1074/jbc.M115.706523 PMC4751403 26663081

[9] Bompard J Rosso A Brizuela L Mebarek S Blum LJ Trunfio-Sfarghiu A-M. Membrane Fluidity as a New Means to Selectively Target Cancer Cells With Fusogenic Lipid Carriers. Langmuir (2020) 36:5134–44. doi: 10.1021/acs.langmuir.0c00262 32338922

[10] Rysman E Brusselmans K Scheys K Timmermans L Derua R Munck S. *De Novo* Lipogenesis Protects Cancer Cells From Free Radicals and Chemotherapeutics by Promoting Membrane Lipid Saturation. Cancer Res (2010) 70:8117–26. doi: 10.1158/0008-5472.CAN-09-3871 20876798

[11] Schug ZT Peck B Jones DT Zhang Q Grosskurth S Alam IS. Acetyl-CoA Synthetase 2 Promotes Acetate Utilization and Maintains Cancer Cell Growth Under Metabolic Stress. Cancer Cell (2015) 27:57–71. doi: 10.1016/j.ccell.2014.12.002 25584894 PMC4297291

[12] Lisec J Jaeger C Zaidi N. Cancer Cell Lipid Class Homeostasis Is Altered Under Nutrient-Deprivation But Stable Under Hypoxia. bioRxiv (2018) 382457. doi: 10.1101/382457 PMC6537432 31138183

[13] Jarc E Petan T. Lipid Droplets and the Management of Cellular Stress. Yale J Biol Med (2019) 92:435–52. PMC6747940 31543707

[14] Wolins NE Quaynor BK Skinner JR Schoenfish MJ Tzekov A Bickel PE. S3-12, Adipophilin, and TIP47 Package Lipid in Adipocytes. J Biol Chem (2005) 280:19146–55. doi: 10.1074/jbc.M500978200 15731108

[15] Schott MB Weller SG Schulze RJ Krueger EW Drizyte-Miller K Casey CA. Lipid Droplet Size Directs Lipolysis and Lipophagy Catabolism in Hepatocytes. J Cell Biol (2019) 218:3320–35. doi: 10.1083/jcb.201803153 PMC6781454 31391210

[16] Abramczyk H Surmacki J Kopeć M Olejnik AK Lubecka-Pietruszewska K Fabianowska-Majewska K. The Role of Lipid Droplets and Adipocytes in Cancer. Raman Imaging of Cell Cultures: MCF10A, MCF7, and MDA-MB-231 Compared to Adipocytes in Cancerous Human Breast Tissue. Analyst (2015) 140:2224–35. doi: 10.1039/C4AN01875C 25730442

[17] Li X Li Y Jiang M Wu W He S Chen C. Quantitative Imaging of Lipid Synthesis and Lipolysis Dynamics in *Caenorhabditis Elegans* by Stimulated Raman Scattering Microscopy. Anal Chem (2019) 91:2279–87. doi: 10.1021/acs.analchem.8b04875 30589537

[18] Paar M Jüngst C Steiner NA Magnes C Sinner F Kolb D. Remodeling of Lipid Droplets During Lipolysis and Growth in Adipocytes. J Biol Chem (2012) 287:11164–73. doi: 10.1074/jbc.M111.316794 PMC3322829 22311986

[19] Sun X Wang M Wang M Yu X Guo J Sun T. Metabolic Reprogramming in Triple-Negative Breast Cancer. Front Oncol (2020) 10:428. doi: 10.3389/fonc.2020.00428 32296646 PMC7136496

[20] Petővári G Dankó T Tőkés A-M Vetlényi E Krencz I Raffay R. *In Situ* Metabolic Characterisation of Breast Cancer and Its Potential Impact on Therapy. Cancers (2020) 12:2492. doi: 10.3390/cancers12092492 PMC7563878 32899149

[21] Lampa M Arlt H He T Ospina B Reeves J Zhang B. Glutaminase Is Essential for the Growth of Triple-Negative Breast Cancer Cells With a Deregulated Glutamine Metabolism Pathway and its Suppression Synergizes With mTOR Inhibition. PloS One (2017) 12:e0185092. doi: 10.1371/journal.pone.0185092 28950000 PMC5614427

[22] Jung SM Hung C-M Hildebrand SR Sanchez-Gurmaches J Martinez-Pastor B Gengatharan JM. Non-Canonical Mtorc2 Signaling Regulates Brown Adipocyte Lipid Catabolism Through SIRT6-Foxo1. Mol Cell (2019) 75:807–822.e8. doi: 10.1016/j.molcel.2019.07.023 31442424 PMC7388077

[23] Yee LD Mortimer JE Natarajan R Dietze EC Seewaldt VL Health M. Insulin, and Breast Cancer: Why Oncologists Should Care About Insulin. Front Endocrinol (Lausanne) (2020) 11:58. doi: 10.3389/fendo.2020.00058 32153503 PMC7045050

[24] Sanderson SM Gao X Dai Z Locasale JW. Methionine Metabolism in Health and Cancer: A Nexus of Diet and Precision Medicine. Nat Rev Cancer (2019) 19:625–37. doi: 10.1038/s41568-019-0187-8 31515518

[25] Cai H Dong L Liu F. Recent Advances in Adipose mTOR Signaling and Function: Therapeutic Prospects. Trends Pharmacol Sci (2016) 37:303–17. doi: 10.1016/j.tips.2015.11.011 PMC4811695 26700098

[26] Yoon M-S. The Role of Mammalian Target of Rapamycin (mTOR) in Insulin Signaling. Nutrients (2017) 9(11):1176. doi: 10.3390/nu9111176 PMC5707648 29077002

[27] Hay N. Interplay Between FOXO, TOR, and Akt. Biochim Biophys Acta (BBA) - Mol Cell Res (2011) 1813:1965–70. doi: 10.1016/j.bbamcr.2011.03.013 PMC3427795 21440577

[28] Shi X Wang J Lei Y Cong C Tan D Zhou X. Research Progress on the PI3K/AKT Signaling Pathway in Gynecological Cancer. Mol Med Rep (2019) 19:4529–35. doi: 10.3892/mmr.2019.10121 PMC6522820 30942405

[29] Kitada M Xu J Ogura Y Monno I Koya D. Mechanism of Activation of Mechanistic Target of Rapamycin Complex 1 by Methionine. Front Cell Dev Biol (2020) 8:715. doi: 10.3389/fcell.2020.00715 32850834 PMC7431653

[30] Zhou Y Zhou Z Peng J Loor JJ. Methionine and Valine Activate the Mammalian Target of Rapamycin Complex 1 Pathway Through Heterodimeric Amino Acid Taste Receptor (TAS1R1/TAS1R3) and Intracellular Ca2+ in Bovine Mammary Epithelial Cells. J Dairy Sci (2018) 101:11354–63. doi: 10.3168/jds.2018-14461 30268610

[31] Costantino A Milazzo G Giorgino F Russo P Goldfine ID Vigneri R. Insulin-Resistant MDA-MB231 Human Breast Cancer Cells Contain a Tyrosine Kinase Inhibiting Activity. Mol Endocrinol (1993) 7:1667–76. doi: 10.1210/mend.7.12.8145772 8145772

[32] Gupta C Tikoo K. High Glucose and Insulin Differentially Modulates Proliferation in MCF-7 and MDA-MB-231 Cells. J Mol Endocrinol (2013) 51:119–29. doi: 10.1530/JME-13-0062 23690508

[33] Wanders D Hobson K Ji X. Methionine Restriction and Cancer Biology. Nutrients (2020) 12(3):684. doi: 10.3390/nu12030684 PMC7146589 32138282

[34] Borrego SL Fahrmann J Datta R Stringari C Grapov D Zeller M. Metabolic Changes Associated With Methionine Stress Sensitivity in MDA-MB-468 Breast Cancer Cells. Cancer Metab (2016) 4:9. doi: 10.1186/s40170-016-0148-6 27141305 PMC4852440

[35] Jeon H Kim JH Lee E Jang YJ Son JE Kwon JY. Methionine Deprivation Suppresses Triple-Negative Breast Cancer Metastasis *In Vitro* and *In Vivo* . Oncotarget (2016) 7:67223–34. doi: 10.18632/oncotarget.11615 PMC5341870 27579534

[36] Morén B Fryklund C Stenkula K. Surface-Associated Lipid Droplets: An Intermediate Site for Lipid Transport in Human Adipocytes? Adipocyte (2020) 9:636–48. doi: 10.1080/21623945.2020.1838684 PMC7595579 33108251

[37] Covington JD Johannsen DL Coen PM Burk DH Obanda DN Ebenezer PJ. Intramyocellular Lipid Droplet Size Rather Than Total Lipid Content Is Related to Insulin Sensitivity After 8 Weeks of Overfeeding. Obes (Silver Spring) (2017) 25:2079–87. doi: 10.1002/oby.21980 PMC5705570 29071793

[38] DeBose-Boyd RA Ye J. SREBPs in Lipid Metabolism, Insulin Signaling, and Beyond. Trends Biochem Sci (2018) 43:358–68. doi: 10.1016/j.tibs.2018.01.005 PMC5923433 29500098

[39] Borrego SL Fahrmann J Hou J Lin D-W Tromberg BJ Fiehn O. Lipid Remodeling in Response to Methionine Stress in MDA-MBA-468 Triple-Negative Breast Cancer Cells. J Lipid Res (2021) 62:100056. doi: 10.1016/j.jlr.2021.100056 33647277 PMC8042402

[40] Murata Y Watanabe T Sato M Momose Y Nakahara T Oka S. Dimethyl Sulfoxide Exposure Facilitates Phospholipid Biosynthesis and Cellular Membrane Proliferation in Yeast Cells. J Biol Chem (2003) 278:33185–93. doi: 10.1074/jbc.M300450200 12771156

[41] Zou K Rouskin S Dervishi K McCormick MA Sasikumar A Deng C. Life Span Extension by Glucose Restriction Is Abrogated by Methionine Supplementation: Cross-Talk Between Glucose and Methionine and Implication of Methionine as a Key Regulator of Life Span. Sci Adv (2020) 6:eaba1306. doi: 10.1126/sciadv.aba1306 32821821 PMC7406366

[42] Weber FL Veach GL Friedman DW. Effects of Insulin and Glucagon on the Uptake of Amino Acids From Arterial Blood by Canine Ileum. Digest Dis Sci (1981) 26:113–8. doi: 10.1007/BF01312226 7006941

[43] Hou J Williams J Botvinick EL Potma EO Tromberg BJ. Visualization of Breast Cancer Metabolism Using Multimodal Nonlinear Optical Microscopy of Cellular Lipids and Redox State. Cancer Res (2018) 78:2503–12. doi: 10.1158/0008-5472.CAN-17-2618 PMC5955854 29535219

[44] Souba WW. Glutamine and Cancer. Ann Surg (1993) 218:715–28. doi: 10.1097/00000658-199312000-00004 PMC1243066 8257221

[45] Wise DR Thompson CB. Glutamine Addiction: A New Therapeutic Target in Cancer. Trends Biochem Sci (2010) 35:427–33. doi: 10.1016/j.tibs.2010.05.003 PMC2917518 20570523

[46] Charidemou E Ashmore T Li X McNally BD West JA Liggi S. A Randomized 3-Way Crossover Study Indicates That High-Protein Feeding Induces *De Novo* Lipogenesis in Healthy Humans. JCI Insight (2019) 4:e124819. doi: 10.1172/jci.insight.124819 PMC6629161 31145699

[47] Muthusamy T Cordes T Handzlik MK You L Lim EW Gengatharan J. Serine Restriction Alters Sphingolipid Diversity to Constrain Tumour Growth. Nature (2020) 586:790–5. doi: 10.1038/s41586-020-2609-x PMC7606299 32788725

[48] Shi L Zheng C Shen Y Chen Z Silveira ES Zhang L. Optical Imaging of Metabolic Dynamics in Animals. Nat Commun (2018) 9:2995. doi: 10.1038/s41467-018-05401-3 30082908 PMC6079036

[49] MDA-MB-231 (ATCC® HTB-26tm) . Available at: https://www.atcc.org/products/all/htb-26.aspx#culturemethod.

[50] Czamara K Majzner K Pacia MZ Kochan K Kaczor A Baranska M. Raman Spectroscopy of Lipids: A Review. J Raman Spectros (2015) 46:4–20. doi: 10.1002/jrs.4607

[51] Jamieson LE Li A Faulds K Graham D. Ratiometric Analysis Using Raman Spectroscopy as a Powerful Predictor of Structural Properties of Fatty Acids. R Soc Open Sci (2018) 5:181483. doi: 10.1098/rsos.181483 30662753 PMC6304136

[52] Deevska GM Nikolova-Karakashian MN. The Expanding Role of Sphingolipids in Lipid Droplet Biogenesis. Biochim Biophys Acta (BBA) - Mol Cell Biol Lipids (2017) 1862:1155–65. doi: 10.1016/j.bbalip.2017.07.008 28743537

[53] Kullback S Leibler RA. On Information and Sufficiency. Ann Math Statistics (1951) 22:79–86. doi: 10.1214/aoms/1177729694

[54] Henne WM Reese ML Goodman JM. The Assembly of Lipid Droplets and Their Roles in Challenged Cells. EMBO J (2018) 37(12):e98947. doi: 10.15252/embj.201898947 29789390 PMC6003646

[55] Benador IY Veliova M Liesa M Shirihai OS. Mitochondria Bound to Lipid Droplets: Where Mitochondrial Dynamics Regulate Lipid Storage and Utilization. Cell Metab (2019) 29:827–35. doi: 10.1016/j.cmet.2019.02.011 PMC6476311 30905670

[56] Cui L Liu P. Two Types of Contact Between Lipid Droplets and Mitochondria. Front Cell Dev Biol (2020) 8:1589. doi: 10.3389/fcell.2020.618322 PMC7769837 33385001

[57] Kitt JP Bryce DA Minteer SD Harris JM. Raman Spectroscopy Reveals Selective Interactions of Cytochrome C With Cardiolipin That Correlate With Membrane Permeability. J Am Chem Soc (2017) 139:3851–60. doi: 10.1021/jacs.7b00238 28221789

[58] Sato ET Martinho H. First-Principles Calculations of Raman Vibrational Modes in the Fingerprint Region for Connective Tissue. Biomed Opt. Express (2018) 9:1728. doi: 10.1364/BOE.9.001728 29675314 PMC5905918

[59] Farber C Li J Hager E Chemelewski R Mullet J Yu. Rogachev A. Complementarity of Raman and Infrared Spectroscopy for Structural Characterization of Plant Epicuticular Waxes. ACS Omega (2019) 4:3700–7. doi: 10.1021/acsomega.8b03675

[60] Khalid M Bora T Ghaithi AA Thukral S Dutta J. Raman Spectroscopy Detects Changes in Bone Mineral Quality and Collagen Cross-Linkage in Staphylococcus Infected Human Bone. Sci Rep (2018) 8:9417. doi: 10.1038/s41598-018-27752-z 29925892 PMC6010429

[61] Da Silva E Bresson S Rousseau D. Characterization of the Three Major Polymorphic Forms and Liquid State of Tristearin by Raman Spectroscopy. Chem Phys Lipids (2009) 157:113–9. doi: 10.1016/j.chemphyslip.2008.11.002 19056367

[62] Podsednik A Jacob A Li LZ Xu HN. Relationship Between Optical Redox Status and Reactive Oxygen Species in Cancer Cells. React Oxyg Species (Apex) (2020) 9:95–108. doi: 10.20455/ros.2020.815 32066994 PMC7025870

[63] Ostrander JH McMahon CM Lem S Millon SR Brown JQ Seewaldt VL. Optical Redox Ratio Differentiates Breast Cancer Cell Lines Based on Estrogen Receptor Status. Cancer Res (2010) 70:4759–66. doi: 10.1158/0008-5472.CAN-09-2572 PMC3826951 20460512

[64] Yun Y-H Bin J Liu D-L Xu L Yan T-L Cao D-S. A Hybrid Variable Selection Strategy Based on Continuous Shrinkage of Variable Space in Multivariate Calibration. Anal Chimica Acta (2019) 1058:58–69. doi: 10.1016/j.aca.2019.01.022 30851854

[65] Hotamisligil GS. Inflammation and Metabolic Disorders. Nature (2006) 444:860–7. doi: 10.1038/nature05485 17167474

[66] Adams WR Mehl B Leiser E Wang M Patton S Throckmorton GA. “Multimodal Nonlinear Optical and Thermal Imaging Platform for Label-Free Characterization of Biological Tissue”. Biophysics (2020). doi: 10.1101/2020.04.06.023820 PMC8044215 33850171

[67] Chen G Deng X. Cell Synchronization by Double Thymidine Block. Bio Protoc (2018) 8:e2994. doi: 10.21769/BioProtoc.2994 PMC6156087 30263905

[68] O’Malley J Kumar R Kuzmin A Pliss A Yadav N Balachandar S. Lipid Quantification by Raman Microspectroscopy as a Potential Biomarker in Prostate Cancer. Cancer Lett (2017) 397:52–60. doi: 10.1016/j.canlet.2017.03.025 28342983 PMC5449194

[69] Zhang L Li C Peng D Yi X He S Liu F. Raman Spectroscopy and Machine Learning for the Classification of Breast Cancers. Spectrochim Acta Part A: Mol Biomol Spectros (2022) 264:120300. doi: 10.1016/j.saa.2021.120300 34455388

[70] Kingma DP Ba J. Adam: A Method for Stochastic Optimization. In: Arxiv:1412.6980 [Cs] (2017). Available at: http://arxiv.org/abs/1412.6980.

[71] Pedregosa F Varoquaux G Gramfort A Michel V Thirion B Grisel O. Scikit-Learn: Machine Learning in Python. J Mach Learn Res (2011) 12:2825–30.

[72] Sgarro A. Informational Divergence and the Dissimilarity of Probability Distributions. Calcolo (1981) 18:293–302. doi: 10.1007/BF02576360

[73] Garcıa-Garcıa D Williamson RC. Divergences and Risks for Multiclass Experiments JMLR: Workshop and Conference Proceedings 2012. 20:1–20.

[74] Velioglu SD Ercioglu E Temiz HT Velioglu HM Topcu A Boyaci IH. Raman Spectroscopic Barcode Use for Differentiation of Vegetable Oils and Determination of Their Major Fatty Acid Composition. J Am Oil Chem Soc (2016) 93:627–35. doi: 10.1007/s11746-016-2808-7

