# Supplementary material for: Imaging Sub-Cellular Methionine and Insulin Interplay in Triple Negative Breast Cancer Lipid Droplet Metabolism
Source: Front Oncol. 2022 Mar 10;12:858017. doi: 10.3389/fonc.2022.858017 (PMC8960266; doi:10.3389/fonc.2022.858017)
Supplement: Supplementary file 1 [file DataSheet_1.pdf]

Imaging Methionine and Insulin Interplay in Lipid Droplet Metabolism in Breast Cancer Cells

Anthony A. Fung, Khang Hoang, Honghao Zha, Derek Chen, Wenxu Zhang, Lingyan Shi*

*Corresponding author. Email: Lingyanshi@ucsd.edu

This PDF file includes:

Figs. S1 to S4
Tables S1

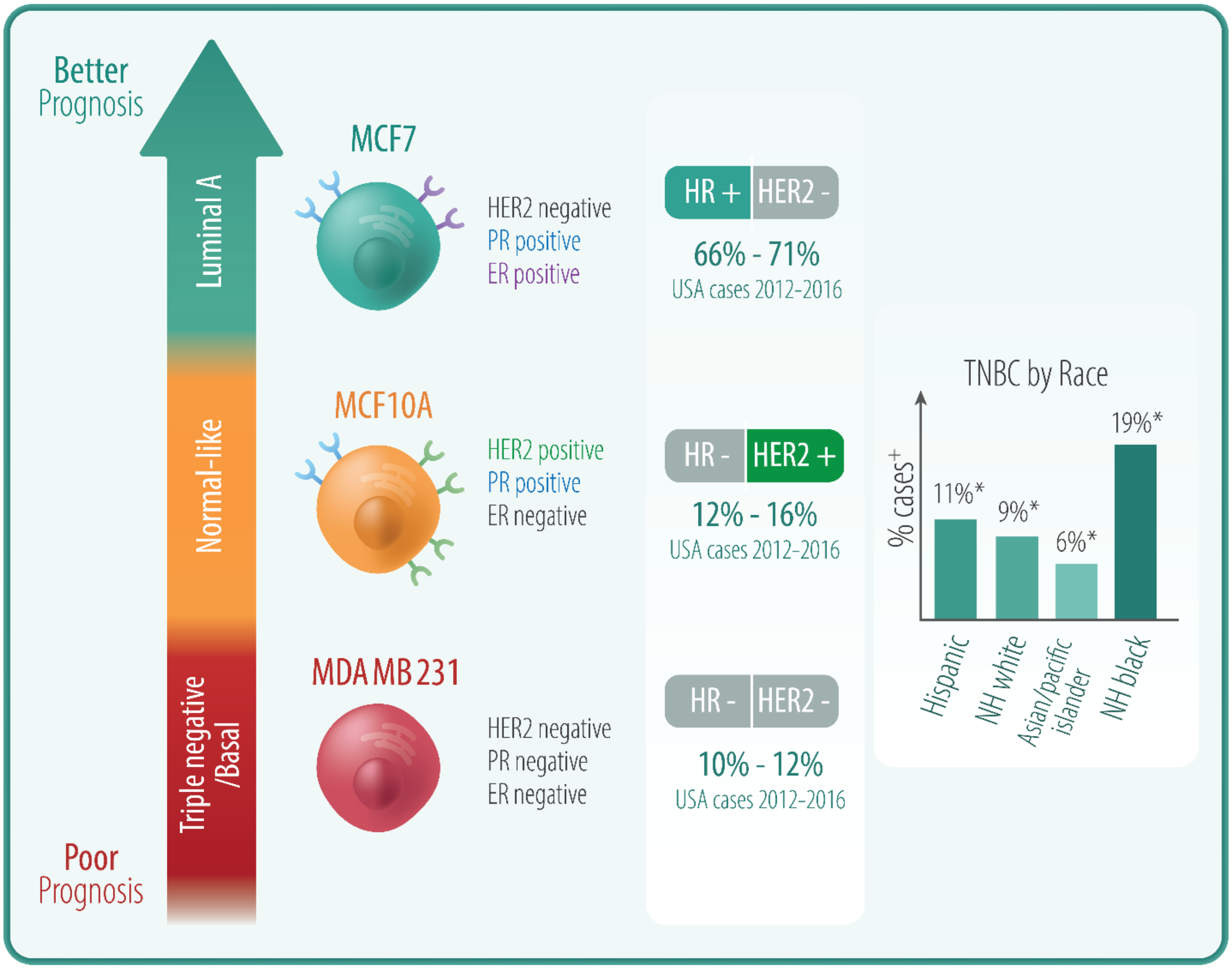

Figure S1. Breast cancer prognosis may be heavily influenced by subtype. Subtypes may be classified by membrane proteins that afford hormone sensitivity but may be further delineated by other aspects of the proteome and metabolic phenotype. TNBC affects non-Hispanic black women more frequently than any other race, but the exact reasons for this discriminatory behavior are not currently known.

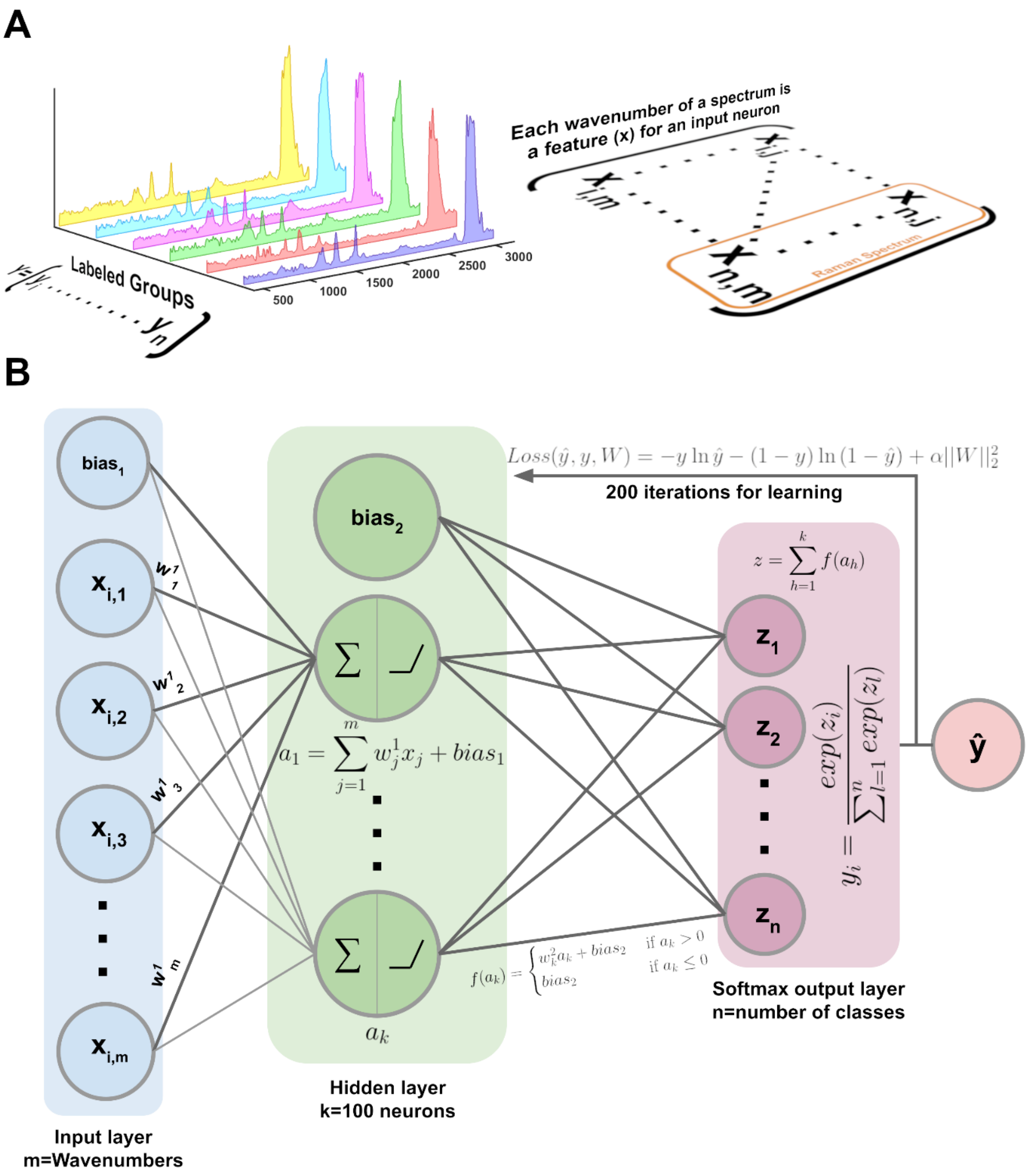

Figure S2. **(A)** Input for the MLP neural network classification model. All n spectra are labeled as strings which are stored in target vector Y with n categorical variables. All spectra contain the same number of features, so that the final input matrix has size n **x** m corresponding to number of spectra, and wavenumber variables, respectively. **(B)** MLP model for multiclass prediction of breast cancer subtypes and metabolic phenotypes. Raman intensities are sent to the input layer, multiplied by a weight vector of the same size as wavenumbers, and summed in each hidden layer neuron. A ReLU activation function determines what value is sent to the output layer, where the hidden layer neuron outputs are summed for each class. Softmax calculates the predicted target class ŷ based on the which z corresponds to the highest probability. The model learns by minimizing cross-entropy loss in which the gradient $W^{i+1} = W^i - \epsilon \nabla Loss_W^i$.

Raman spectra from 450cm^{-1} to 3150cm^{-1} share very similar patterns with previous studies aimed at high throughput single cell analysis of lipid droplets, with the more aggressive triple negative subtype having a relatively weaker lipid presence [17]. These spectra were then fed into a simple

ReLu neural network for classification. The results demonstrate that Raman spectroscopy of lipid droplets achieved a label-free classification accuracy of 82% with 200 iterations in a 10-fold cross validation shown in Figure 6. Of note is the poorer distinction between the MDA-MB-231 cells that were supplied excess methionine (denoted by "high Met") and those that were not. This indicates that TNBC may potentially be less demanding of methionine or less responsive to it.

Confusion Matrix

Confusion matrix for Neural Network (showing number of instances)

	Predicted: MBMDA231 D2O	MBMDA231 D2O high Met	MCF10A D2O	MCF10A D2O high Met	MCF7 D2O	MCF7 D2O high Met	Σ
Actual: MBMDA231 D2O	10	1	0	0	0	0	11
MBMDA231 D2O high Met	4	4	0	0	0	0	8
MCF10A D2O	0	0	5	1	0	1	7
MCF10A D2O high Met	0	0	1	5	0	1	7
MCF7 D2O	0	0	0	0	9	0	9
MCF7 D2O high Met	0	0	0	0	0	8	8
Σ	14	5	6	6	9	10	50

Figure S3. Confusion matrix illustrating the 10-fold cross validation accuracy of the ReLU neural network. 100 neurons in the hidden layer and 200 iterations with the Adam solver achieved AUC: 0.9811, CA: 0.82, F1: 0.812, Precision: 0.823, Recall: 0.82. Classification between dietary methionine concentrations in TNBC cell line MDA-MB-231 was the poorest.

Table S1. 2-way ANOVA of figure 4B shows significant interaction term Insulin*Methionine in TNBC.

ANOVA MDA-MB-231 CH_2:CH_3

	Sum Sq.	d.f.	Mean Sq.	F	Prob>F
**Insulin**	0.023695	2	0.011848	2.841	0.0741
**Methionine**	0.084655	1	0.084655	20.2999	**0.0001**
**Insulin*Methionine**	0.034647	2	0.017324	4.1541	**0.0256**
**Error**	0.12511	30	0.0041702		
**Total**	0.2681	35			

Table S2. 2-way ANOVA of figure 5A-B. Relative lipid metabolism characterized by the ratio of de novo synthesized and total lipids was not significantly sensitive to methionine concentration in normal-like breast cells, while there is a significant interaction term Insulin*Methionine in TNBC. In both cell types, methionine concentration was the significant independent variable in terms of relative lipid and protein synthesis.

A

MCF-10A $CD_L:CD_P$

	Sum Sq.	d.f.	Mean Sq.	F	Prob>F
Insulin	0.008009	2	0.0040045	0.36443	0.69523
Methionine	0.061128	1	0.061128	5.563	**0.019688**
Insulin*Methionine	0.0031567	2	0.0015784	0.14364	0.86633
Error	1.5823	144	0.010988		
Total	1.6546	149			

MCF-10A $CD_L:CH_2$

	Sum Sq.	d.f.	Mean Sq.	F	Prob>F
Insulin	0.0010429	2	0.0005215	15.0812	**1.13E-06**
Methionine	3.46E-06	1	3.46E-06	0.10001	0.75227
Insulin*Methionine	3.34E-05	2	1.67E-05	0.48336	0.61771
Error	0.004979	144	3.46E-05		
Total	0.0060588	149			

B

MDA-MB-231 $CD_L:CD_P$

	Sum Sq.	d.f.	Mean Sq.	F	Prob>F
Insulin	0.046629	2	0.023315	1.9588	0.14477
Methionine	0.25799	1	0.25799	21.675	**7.27E-06**
Insulin*Methionine	0.038589	2	0.019295	1.621	0.20128
Error	1.714	144	0.011903		
Total	2.0572	149			

MDA-MB-231 $CD_L:CH_2$

	Sum Sq.	d.f.	Mean Sq.	F	Prob>F
Insulin	0.04666	2	0.0065004	16.2293	**4.40E-07**
Methionine	0.0031618	1	0.0090534	22.6031	**4.78E-06**
Insulin*Methionine	0.0008114	2	0.0013273	3.3137	**0.039174**
Error	0.059362	144	0.0004005		
Total	0.10999	149			

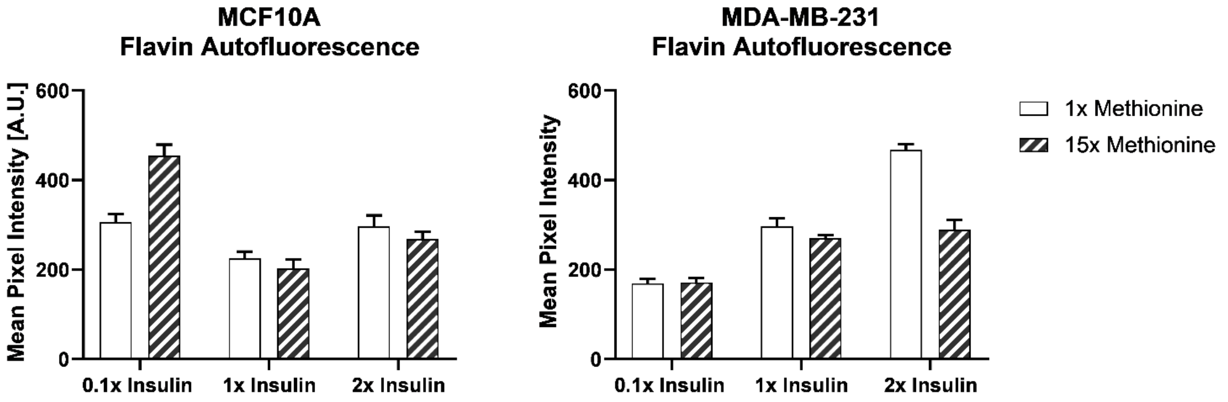

Figure S4. Quantitative summary of flavin autofluorescence from TPF image analysis. Excess methionine generally decreased flavin autofluorescence, whereas higher insulin concentrations generally increased flavin autofluorescence.
